# Moderate overexpression of *PROTON GRADIENT REGULATION 5* improves photosynthetic performance and plant growth under fluctuating low light in *Arabidopsis thaliana*

**DOI:** 10.1111/tpj.71120

**Published:** 2026-09-28

**Authors:** Keiichiro Tanigawa, Hiromasa Kodama, Yuki Okegawa, Toshiharu Shikanai, Wataru Yamori

**Affiliations:** ^1^ Graduate School of Agricultural and Life Sciences The University of Tokyo Nishitokyo Tokyo Japan; ^2^ Institute of Plant Science and Resources, Okayama University Kurashiki 710‐0046 Okayama Japan; ^3^ Graduate School of Science Kyoto University Sakyo‐ku 606‐8502 Kyoto Japan

**Keywords:** Arabidopsis thaliana, cyclic electron transport, fluctuating light, PGR5, photoprotection, photosynthetic induction, photosystem I

## Abstract

Cyclic electron transport around photosystem I (PSI) plays an important role in regulating photosynthesis by balancing ATP/NADPH production and maintaining PSI function under fluctuating light. Although PGR5‐dependent photosynthetic regulation has been extensively studied under high or fluctuating light, its physiological significance under prolonged low irradiance interrupted by transient increases in light remains less well understood. Here, we analyzed *Arabidopsis thaliana* lines with different levels of PGR5 accumulation to investigate how PGR5 abundance influences photosynthetic regulation and plant performance under controlled fluctuating light conditions. Moderate PGR5 accumulation was associated with faster photosynthetic induction, improved PSI redox regulation, and greater biomass accumulation under the 100 μmol photons m^−2^ s^−1^ fluctuating light regime. In contrast, the absence of PGR5 severely impaired photosynthetic performance, particularly under the 600 μmol photons m^−2^ s^−1^ fluctuating light regime, whereas excessively high PGR5 accumulation did not provide additional physiological benefits. These results indicate that photosynthetic responses varied according to PGR5 abundance, but the relationship was not simply proportional across all genotypes. Our findings demonstrate that maintaining an appropriate level of PGR5 is important for efficient photosynthetic regulation under fluctuating light conditions. This study provides new physiological insights into the role of PGR5 under dynamic light environments and establishes a framework for future studies aimed at optimizing photosynthesis under naturally fluctuating irradiance.

## Significance Statement

Moderate increase in the PGR5 improves plant productivity, whereas excessive PGR5 accumulation impaired photosynthetic performance and reduced plant growth. Therefore, fine‐tuning PGR5 expression to achieve optimal PGR5 accumulation may be an effective strategy for improving crop productivity.

## INTRODUCTION

Photosynthesis is the fundamental process by which light energy is converted into chemical energy, sustaining almost all life on Earth (Yamori et al., 2026). In chloroplasts, light energy drives the production of ATP and NADPH, which serve as essential energy sources for the carbon‐fixing reactions of the Calvin–Benson cycle (Yamori & Shikanai, 2016). The primary electron transport pathway, known as linear electron transport (LET), involves the transfer of electrons from water via photosystem II (PSII), plastoquinone (PQ), the cytochrome *b*_6_ *f* complex (Cyt *b*_6_ *f*), plastocyanin (PC), and photosystem I (PSI), ultimately reducing NADP^+^ to NADPH. Coupled with LET, protons are translocated from the stroma into the thylakoid lumen, generating a proton motive force (*pmf*) across the thylakoid membrane that drives ATP synthesis (Allen, 2002).

Despite the central role of LET, it does not satisfy the stoichiometric requirements of the Calvin–Benson cycle, which demands an ATP/NADPH ratio of approximately 1.5 (Allen, 2003). LET alone typically yields a ratio of about 1.29 due to the fixed stoichiometry of proton translocation and ATP synthesis (Hahn et al., 2018). To compensate for this imbalance and to regulate the redox poise of the electron transport chain, plants employ an alternative pathway known as cyclic electron transport (CET) around PSI (Yamori & Shikanai, 2016). CET functions to generate additional *pmf* and ATP without the concomitant production of NADPH, thereby restoring the ATP/NADPH balance and providing photoprotective benefits under fluctuating environmental conditions (Shikanai, 2007).

In angiosperms, CET is mediated through two partially redundant pathways: one dependent on the PROTON GRADIENT REGULATION 5 (PGR5) and the other involving the chloroplast NADH dehydrogenase‐like (NDH) complex (DalCorso et al., 2008; Endo et al., 1998; Munekage et al., 2002). Among these, the PGR5‐dependent pathway is considered the primary route in most C_3_ plants under normal physiological conditions. Mutants deficient in PGR5 (*pgr5*) exhibit severe photosynthetic defects, including impaired *pmf* formation, reduced non‐photochemical quenching (NPQ), and increased photoinhibition of PSI, particularly under fluctuating or high light conditions (Kobayashi et al., 2024; Munekage et al., 2002; Suorsa et al., 2012; Yamori et al., 2016). These phenotypes underline the critical role of PGR5 in maintaining redox balance and protecting the photosynthetic machinery under variable light environments (Tikkanen et al., 2014; Yamori & Shikanai, 2016).

The molecular function of PGR5, however, remains a subject of ongoing investigation. Previously, the most widely accepted hypothesis was that PGR5 does not itself function as an electron carrier but rather plays a regulatory role in controlling the flow of electrons from PSI to the PQ pool, possibly via interaction with PGRL1. The PGR5‐PGRL1 complex has been proposed to serve as a ferredoxin‐plastoquinone reductase (FQR), catalyzing the reduction of PQ using electrons from ferredoxin (Hertle et al., 2013). However, recent studies are leading to a re‐evaluation of this long‐held hypothesis. Rühle et al. (2021) suggested that PGR5 likely functions independently. The role of PGRL1 is related to maintaining the stability of PGR5, specifically by potentially preventing its degradation by another protein, PGRL2. Recent evidence also indicates that the activity of this complex may be tightly regulated by the redox state of the stroma and post‐translational modifications, such as thiol modulation (Okegawa et al., 2020).

The physiological importance of CET is particularly pronounced under fluctuating light (FL) conditions, which are prevalent in natural environments due to sunflecks, cloud cover, and leaf movements (Pearcy, 1990; Yamori, 2016). Under such conditions, PSI is especially vulnerable to photoinhibition caused by over‐reduction of its acceptor side. PGR5‐dependent CET has been shown to prevent PSI over‐reduction by sustaining an adequate *pmf*, which in turn slows down electron flow into PSI through downregulation of the Cyt *b*_6_ *f* complex (Suorsa et al., 2012). This mechanism, known as photosynthetic control, helps maintain PSI activity and protects against photodamage (Kono et al., 2025). In *pgr5* mutants, the loss of CET capacity leads to rapid over‐reduction of PSI and subsequent photoinhibition during transitions from low to high light, thereby compromising overall photosynthetic performance and growth (Tikkanen et al., 2014; Yamori et al., 2016).

In addition to its photoprotective function, PGR5‐mediated CET is implicated in optimizing photosynthetic induction, a process in which the photosynthetic apparatus gradually activates upon transition from dark or low light to high light. Efficient CET facilitates the establishment of *pmf* and rapid generation of ATP, which are required for activating carbon fixation enzymes and initiating photoprotective responses such as NPQ (Suorsa et al., 2012). Delayed or impaired CET, as observed in *pgr5* mutants, often results in sluggish induction of photosynthesis (Okegawa et al., 2020), reduced energy utilization efficiency, and compromised carbon gain during short light transients.

While the role of PGR5 under high and fluctuating light has been extensively studied, its function under low light conditions remains less understood. Given that natural light environments often include prolonged periods of low irradiance interspersed with brief sunflecks, understanding how PGR5 contributes to photosynthetic performance and plant growth in low light is crucial. Moreover, most studies to date have focused on either loss‐of‐function mutants or lines with extreme overexpression of *PGR5*, which can result in pleiotropic effects such as altered levels of other thylakoid proteins and aberrant growth phenotypes (Long et al., 2008; Munekage et al., 2008; Okegawa et al., 2007). These limitations hinder a nuanced understanding of how PGR5 protein levels quantitatively affect CET activity and downstream physiological responses.

To address these gaps, it is important to investigate a range of transgenic lines with graded *PGR5* expression levels, allowing the dissection of PGR5's dose‐dependent effects on CET, photosynthetic induction, and plant fitness under varying light environments. Such studies will not only clarify the functional threshold and optimal range of PGR5 activity but also offer insights into how CET contributes to light acclimation strategies in plants. Ultimately, these findings could inform approaches to enhance photosynthetic efficiency and stress resilience in crops by fine‐tuning CET capacity through targeted genetic or biochemical interventions.

## RESULTS

### PGR5 protein abundance differs among the transgenic lines without altering the accumulation of other major photosynthetic proteins

To characterize the experimental materials used in this study, we first quantified the abundance of PGR5 protein in the WT, *pgr5‐5* mutant, and four PGR5 overexpression lines (Figure 1b,c). These plants were cultivated at a temperature of 23°C, a relative humidity of 60%, and a 10‐h light/14‐h dark cycle with white light at 120 μmol photons m^−2^ s^−1^ for 6 weeks.

**Figure 1:**
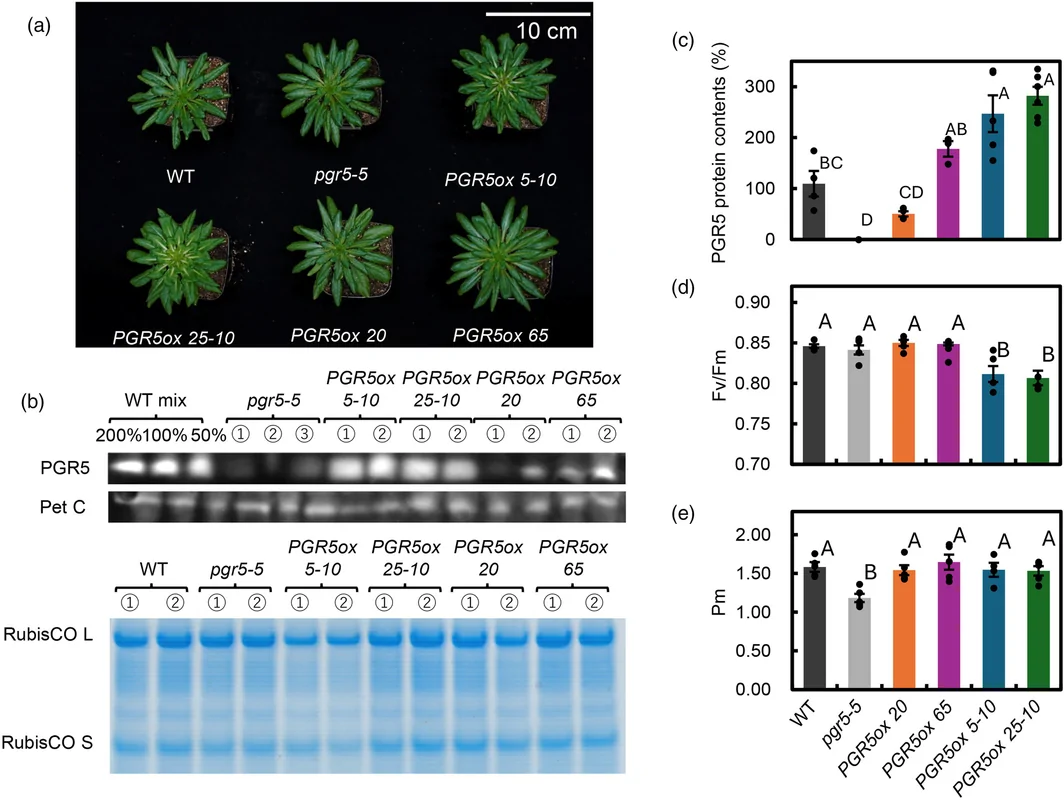
Growth, protein accumulation, steady‐state chlorophyll fluorescence and P700 redox state in WT, *pgr5‐5*, *PGR5ox 20*, *PGR5ox 65*, *PGR5ox 5‐10* and *PGR5ox 25‐10*. (a) Plants grown for 6 weeks under short‐day conditions (10 h light/14 h dark) at 120 μmol photons m^−2^ s^−1^. (b) Immunoblot analysis of PGR5 and PetC proteins and SDS‐PAGE analysis of Rubisco proteins. Sample loading was normalized on a chlorophyll basis. (c) Relative PGR5 protein abundance, with WT set to 100%. (d) Fv/Fm. (e) Pm. Data are means ± SE (n ≥ 3 for c; n ≥ 5 for d and e). Individual data points are shown. Different letters indicate significant differences among genotypes (one‐way ANOVA followed by Tukey's HSD test, *P* < 0.05).

As expected, the *pgr5‐5* mutant accumulated only a trace amount of PGR5 protein, whereas the overexpression lines exhibited different levels of PGR5 accumulation. Among these, PGR5ox 65 accumulated approximately twice as much PGR5 as WT, while PGR5ox 5–10 and PGR5ox 25–10 accumulated approximately threefold more PGR5 protein. In contrast, PGR5ox 20 contained approximately half the PGR5 level of WT, probably owing to co‐suppression (Figure 1b,c). Together, these lines provided a graded series of PGR5 expression levels for subsequent physiological analyses.

To determine whether altered PGR5 expression affected the accumulation of other major photosynthetic proteins, we quantified the Cyt *b*_*6*_ *f* subunit PetC and Rubisco. Neither protein differed significantly among the genotypes (Figure 1b, Figure S1A,B), indicating that changes in PGR5 abundance did not substantially alter the accumulation of these photosynthetic components.

We next examined two commonly used photosynthetic electron transport parameters after dark adaptation. Fv/Fm was significantly lower in the two highest PGR5‐expressing lines (PGR5ox 5‐10 and PGR5ox 25‐10) than in the other genotypes (Figure 1d). In contrast, the maximum photo‐oxidizable P700 signal (Pm) was lowest in the *pgr5‐5* mutant (Figure 1e). These results indicate that altering PGR5 abundance affected photosynthetic characteristics in a genotype‐dependent manner, while leaving the abundance of PetC and Rubisco largely unchanged.

### Photosynthetic induction responses under the 100 μmol photons m^−2^ s^−1^ induction protocol

To determine how PGR5 abundance affects photosynthetic electron transport regulation during photosynthetic induction, we analyzed chlorophyll fluorescence‐ and P700‐derived parameters using the 100 μmol photons m^−2^ s^−1^ induction protocol (Figure 2, Figures [Link], [Link], [Link], [Link] and [Link], [Link], [Link]). Parameters related to PSII [Y(II), NPQ, 1 − qL and ETRII] and PSI [Y(I), Y(ND), Y(NA) and ETRI] were quantified during the induction response. To distinguish presence–absence effects from potential dosage‐dependent effects, relationships were analyzed both including (Figure 2) and excluding the *pgr5‐5* mutant (Figures [Link], [Link], [Link]).

**Figure 2:**
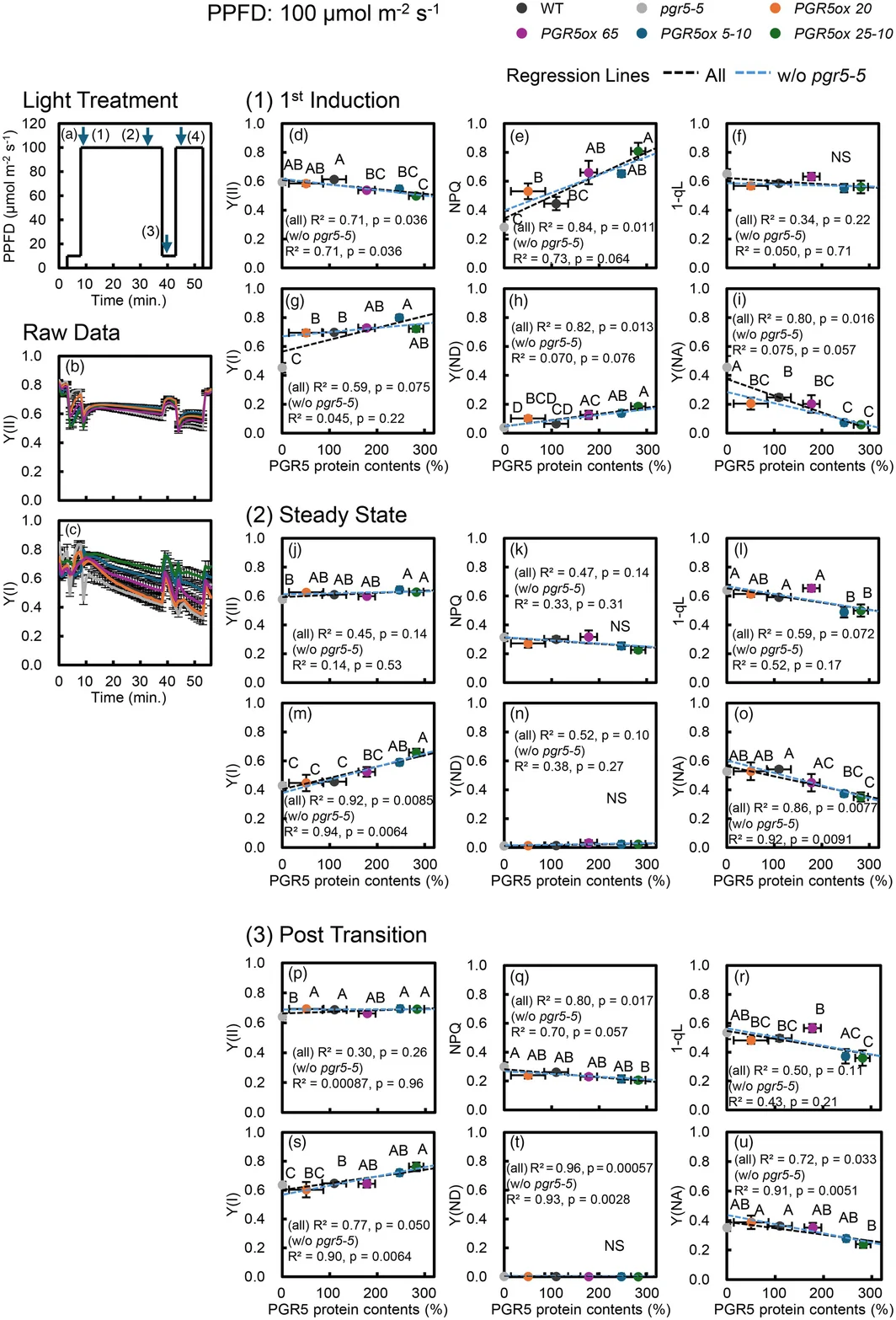
Chlorophyll fluorescence and P700 analysis of a photosynthetic induction response in WT, *pgr5‐5*, *PGR5ox 20*, *PGR5ox 65*, *PGR5ox 5‐10*, and *PGR5ox 25‐10* under the 100 μmol photons m^−2^ s^−1^ induction protocol. (a) Light regime used for the 100 μmol photons m^−2^ s^−1^ induction protocol. (b) Time course of Y(II). (c) Time course of Y(I). (d–i) Relationships between PGR5 protein abundance and Y(II), NPQ, 1 − qL, Y(I), Y(ND), and Y(NA), respectively, during the first induction phase. (j–o) Corresponding relationships during the steady‐state phase. (p–u) Corresponding relationships during the post‐transition phase. Data are means ± SE (n ≥ 4). Different letters indicate significant differences among genotypes (one‐way ANOVA followed by Tukey's HSD test, *P* < 0.05). Regression lines were fitted using the least‐squares method; black lines represent the regression across all genotypes, and blue lines represent the regression excluding the pgr5‐5 mutant. Corresponding analyses excluding the pgr5‐5 mutant are presented in Figures [Link], [Link], [Link].

Overall time‐course measurements showed that the *pgr5‐5* mutant differed markedly from the other genotypes, whereas the overexpression lines exhibited more subtle differences (Figure 2b,c, Figures [Link], [Link]). Among the overexpression lines, the PGR5ox 65 line frequently displayed distinct responses compared with the highest‐expressing lines (PGR5ox 5‐10 and PGR5ox 25‐10), suggesting that moderate PGR5 accumulation affects photosynthetic regulation differently from excessive PGR5 accumulation.

To facilitate comparison among genotypes, four representative phases of the induction response were analyzed: The first induction, the steady‐state phase, the post‐transition phase following the reduction in irradiance, and the second induction (Figure 2a).

#### PSII‐related parameters

During the first induction phase, Y(II) and ETRII were highest in the *pgr5‐5* mutant, whereas the overexpression lines showed only modest differences (Figure 2d, Figure S5A). NPQ increased with increasing PGR5 abundance and reached the highest values in PGR5ox 5‐10 and PGR5ox 25‐10 (Figure 2e). When the *pgr5‐5* mutant was excluded, the relationship between PGR5 abundance and NPQ remained significant, whereas relationships for Y(II) and ETRII became weaker (Figures S14D,E and S16A). Similar trends were observed during the steady‐state and second induction phases (Figure 2j–u, Figures [Link], [Link], [Link]).

#### PSI‐related parameters

PSI‐related parameters showed clearer relationships with PGR5 abundance than PSII parameters did. During the first induction phase, Y(I), Y(ND), and ETRI increased, whereas Y(NA) decreased with increasing PGR5 abundance (Figure 2g–i, Figure S5B). These relationships remained evident after exclusion of the *pgr5‐5* mutant, although the strength of the correlations varied among parameters (Figures S14G–I and S16B). Comparable trends were observed during the steady‐state and second induction phases (Figure 2m–u, Figures [Link], [Link], [Link]).

Overall, PSI‐related parameters showed more consistent associations with PGR5 abundance than PSII‐related parameters under the 100 μmol photons m^−2^ s^−1^ induction protocol. Although the *pgr5‐5* mutant exhibited the largest differences, several PSI‐related responses remained associated with PGR5 abundance even when the mutant was excluded from the analysis.

### Photosynthetic induction responses under the 600 μmol photons m^−2^ s^−1^ induction protocol

To examine the effects of PGR5 abundance under stronger irradiance, photosynthetic electron transport parameters were analyzed using the 600 μmol photons m^−2^ s^−1^ induction protocol (Figure 3, Figures [Link], [Link], [Link] and [Link], [Link], [Link]). As for the 100 μmol photons m^−2^ s^−1^ induction protocol, analyses were performed both including and excluding the *pgr5‐5* mutant to distinguish presence–absence effects from potential dosage‐dependent responses.

**Figure 3:**
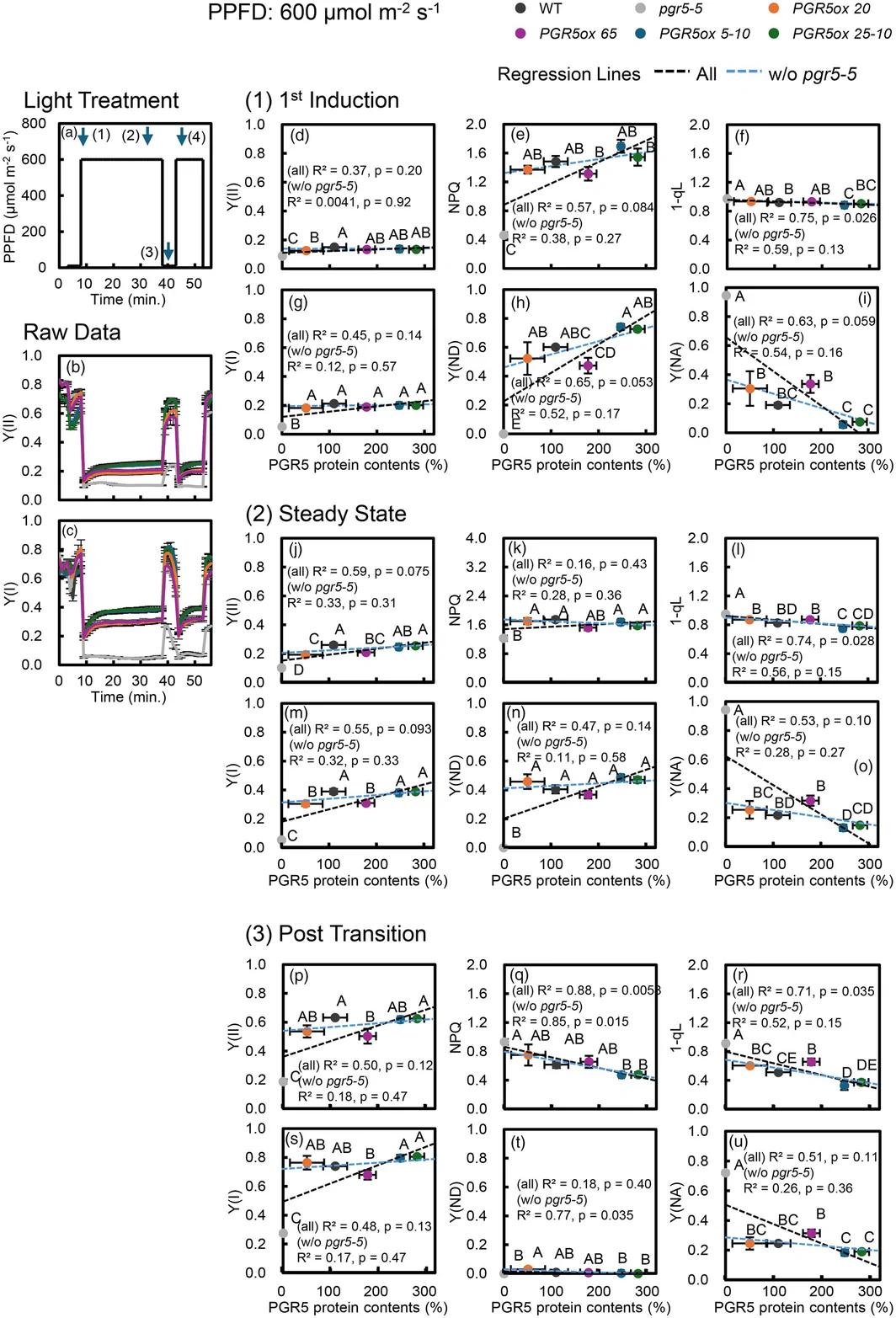
Chlorophyll fluorescence and P700 analysis of a photosynthetic induction response in WT, *pgr5‐5*, *PGR5ox 20*, *PGR5ox 65*, *PGR5ox 5‐10*, and *PGR5ox 25‐10* under the 600 μmol photons m^−2^ s^−1^ induction protocol. (a) Light regime used for the 600 μmol photons m^−2^ s^−1^ induction protocol. (b) Time course of Y(II). (c) Time course of Y(I). (d–i) Relationships between PGR5 protein abundance and Y(II), NPQ, 1 − qL, Y(I), Y(ND), and Y(NA), respectively, during the first induction phase. (j–o) Corresponding relationships during the steady‐state phase. (p–u) Corresponding relationships during the post‐transition phase. Data are means ± SE (n ≥ 4). Different letters indicate significant differences among genotypes (one‐way ANOVA followed by Tukey's HSD test, *P* < 0.05). Regression lines were fitted using the least‐squares method; black lines represent the regression across all genotypes, and blue lines represent the regression excluding the pgr5‐5 mutant. Corresponding analyses excluding the *pgr5‐5* mutant are presented in Figures [Link], [Link], [Link].

Overall, the *pgr5‐5* mutant showed markedly impaired photosynthetic electron transport performance compared with all other genotypes, whereas differences among the overexpression lines were generally much smaller (Figure 3b,c, Figure S6).

#### PSII‐related parameters

During the first induction phase, Y(II), NPQ, and ETRII were significantly lower in the *pgr5‐5* mutant than in the other genotypes (Figure 3d,e, Figure S8A). Similar differences were observed during the steady‐state and second induction phases (Figure 3j,k,p,q, Figure S8). When the *pgr5‐5* mutant was excluded from the analysis, most relationships between PGR5 abundance and these PSII‐related parameters were no longer significant (Figures S17D–E and S19A).

#### PSI‐related parameters

PSI‐related parameters were also strongly affected by the absence of PGR5. The *pgr5‐5* mutant consistently exhibited lower Y(I), Y(ND), and ETRI, together with higher Y(NA), than the other genotypes throughout the induction protocol (Figure 3g–i,m–o,s–u). Among the remaining genotypes, several PSI‐related parameters continued to show significant relationships with PGR5 abundance after exclusion of the *pgr5‐5* mutant, although these relationships were weaker than those observed under the FL100_10 induction protocol (Figures S17H,I,O,S–U and S19F).

Overall, these results indicate that the absence of PGR5 had a much larger effect on photosynthetic regulation under the 600 μmol photons m^−2^ s^−1^ induction protocol than variation in PGR5 abundance among the overexpression lines. Although some PSI‐related parameters remained associated with PGR5 abundance, most responses under this condition reflected a clear presence–absence effect.

### Plant growth and photosynthetic electron transport traits after long‐term light treatments

To determine whether differences in photosynthetic regulation were associated with plant growth, plants were cultivated for three weeks under four long‐term light regimes (FL100_10, CL100_10, FL600_10, and CL600_10) before growth and photosynthetic electron transport traits were evaluated (Figures 4 and 5, Figures S20 and S21).

**Figure 4:**
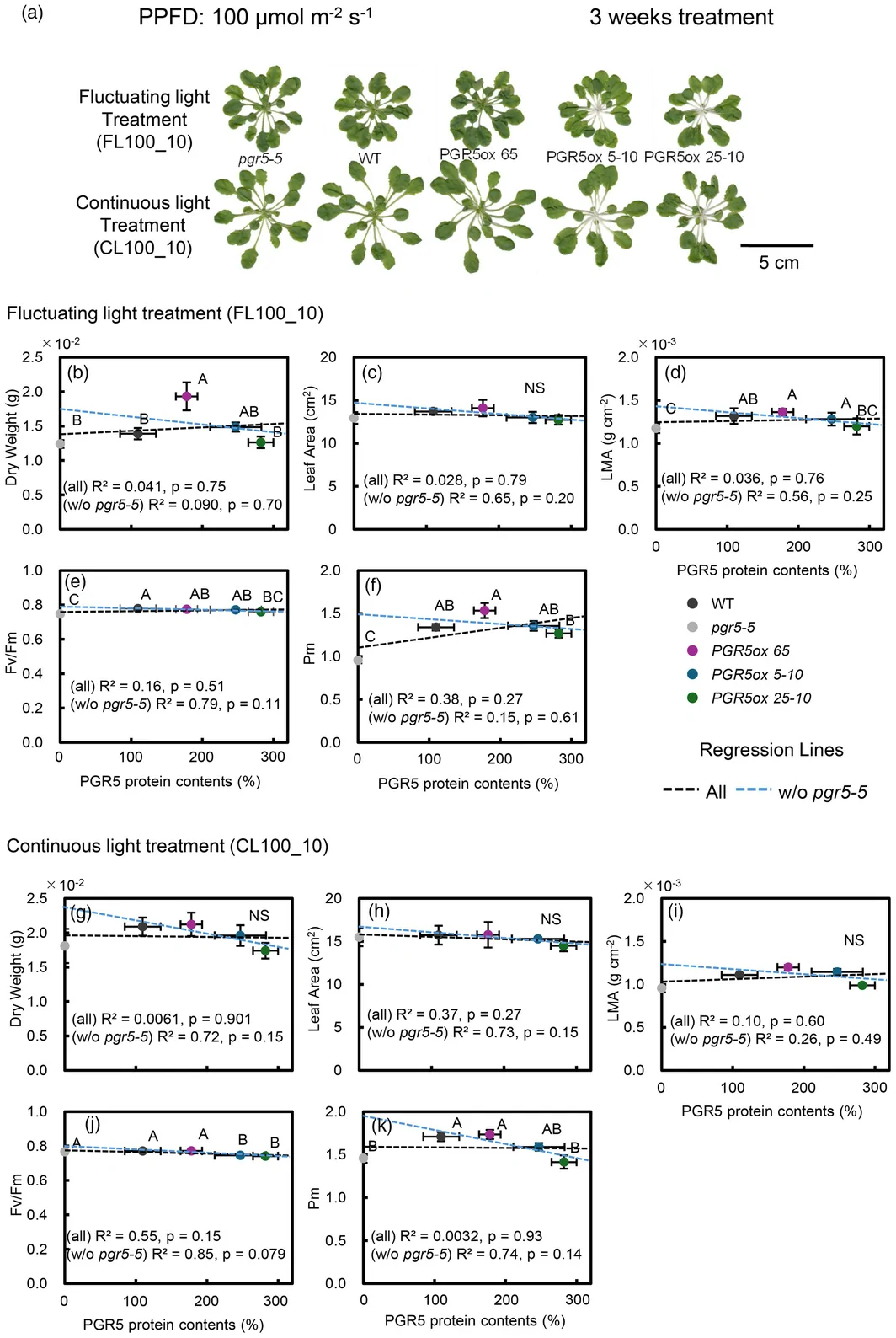
Plant Phenotype and growth analysis, chlorophyll fluorescence and P700 redox state under 3 weeks of FL100_10 and CL100_10 treatment in WT, *pgr5‐5*, *PGR5ox 65*, *PGR5ox 5‐10*, and *PGR5ox 25‐10*. (a) Representative plants after 3 weeks of treatment. Upper panels show plants grown under FL100_10 and lower panels show plants grown under CL100_10. (b–f) Relationships between PGR5 protein abundance and shoot dry weight, leaf area, LMA, Fv/Fm, and Pm, respectively, under FL100_10 conditions. (g–k) Corresponding relationships under CL100_10 conditions. Data are means ± SE (n = 4). Different letters indicate significant differences among genotypes (one‐way ANOVA followed by Tukey's HSD test, *P* < 0.05). Regression lines were fitted using the least‐squares method; black lines represent the regression across all genotypes, and blue lines represent the regression excluding the pgr5‐5 mutant. Corresponding analyses excluding the *pgr5‐5* mutant are presented in Figure S20.

**Figure 5:**
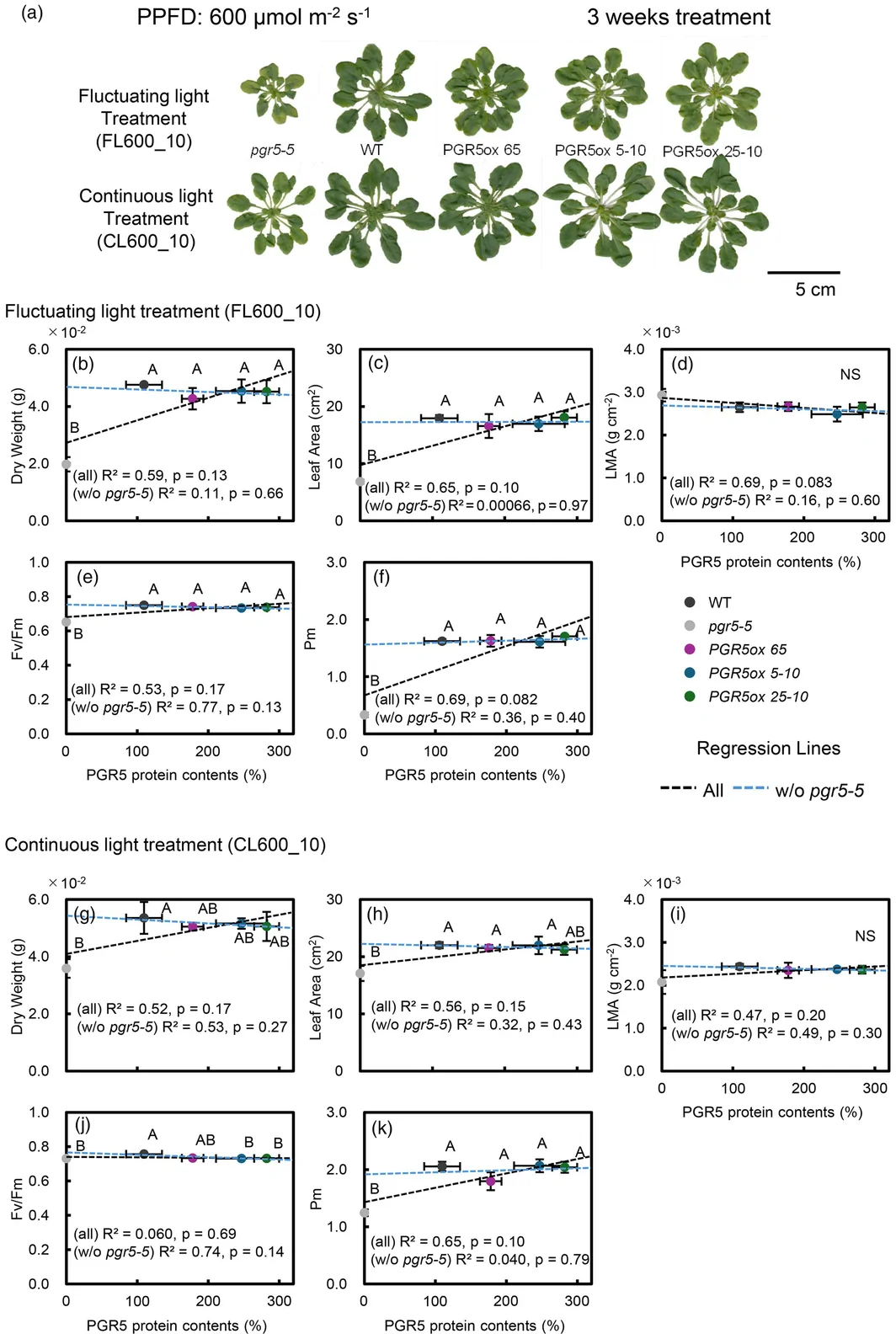
Plant Phenotype and growth analysis, chlorophyll fluorescence and P700 redox state under 3 weeks of FL600_10 and CL600_10 treatment in WT, *pgr5‐5*, *PGR5ox 65*, *PGR5ox 5‐10*, and *PGR5ox 25‐10*. (a) Representative plants after 3 weeks of treatment. Upper panels show plants grown under FL600_10 and lower panels show plants grown under CL600_10. (b–f) Relationships between PGR5 protein abundance and shoot dry weight, leaf area, LMA, Fv/Fm, and Pm, respectively, under FL600_10 conditions. (g–k) Corresponding relationships under CL600_10 conditions. Data are means ± SE (n = 4). Different letters indicate significant differences among genotypes (one‐way ANOVA followed by Tukey's HSD test, *P* < 0.05). Regression lines were fitted using the least‐squares method; black lines represent the regression across all genotypes, and blue lines represent the regression excluding the pgr5‐5 mutant. Corresponding analyses excluding the *pgr5‐5* mutant are presented in Figure S21.

In the FL100_10 treatment, plants were exposed to alternating 10‐min periods of 10 and 100 μmol photons m^−2^ s^−1^. In the corresponding CL100_10 treatment, plants received the same total duration at each irradiance without fluctuations: 2.5 h at 10 μmol photons m^−2^ s^−1^, followed by 5 h at 100 μmol photons m^−2^ s^−1^, and another 2.5 h at 10 μmol photons m^−2^ s^−1^. Similarly, in the FL600_10 treatment, plants were exposed to alternating 10‐min periods of 10 and 600 μmol photons m^−2^ s^−1^, while the CL600_10 treatment provided the same total light duration in a non‐fluctuating manner. Thus, each FL treatment and its corresponding CL counterpart had an identical daily light integral and average irradiance, differing only in the temporal pattern of light exposure.

To distinguish presence–absence effects from potential relationships with PGR5 abundance, analyses were performed both including and excluding the *pgr5‐5* mutant.

#### Growth responses under FL100_10 and CL100_10

Under FL100_10, the PGR5ox 65 line produced significantly greater shoot dry weight than the other genotypes, whereas the remaining overexpression lines did not differ significantly from WT (Figure 4b). In contrast, no significant differences in dry weight were detected among genotypes under CL100_10 (Figure 4g).

Leaf area did not differ significantly among genotypes under either FL100_10 or CL100_10 (Figure 4c,h), indicating that differences in shoot biomass under FL100_10 were not associated with changes in leaf expansion.

LMA showed a similar pattern. The highest LMA was observed in PGR5ox 65 under FL100_10, whereas no significant differences were detected among genotypes under CL100_10 (Figure 4d,i).

Photosynthetic electron transport parameters measured after the growth treatments also differed among genotypes. Under FL100_10, Fv/Fm was lowest in the *pgr5‐5* mutant, whereas under CL100_10 the two highest‐expressing lines (PGR5ox 5–10 and PGR5ox 25–10) exhibited significantly lower Fv/Fm than the other genotypes (Figure 4e,j).

Similarly, Pm was highest in PGR5ox 65 under FL100_10 and, while the *pgr5‐5* mutant exhibited the lowest values under both FL100_10 and CL100_10 (Figure 4f,k).

Regression analyses indicated that several growth and photosynthetic traits were associated with PGR5 abundance under FL100_10. However, these relationships became weaker after exclusion of the *pgr5‐5* mutant, indicating that some responses primarily reflected the absence of PGR5, whereas others remained associated with differences in PGR5 abundance among the overexpression lines (Figure S20).

#### Growth responses under FL600_10 and CL600_10

Growth responses under the FL600_10 and CL600_10 regimes differed markedly from those observed under FL100_10.

Under both FL600_10 and CL600_10, the *pgr5‐5* mutant showed significantly lower shoot dry weight and leaf area than all other genotypes (Figure 5b,c,g,h). In contrast, no significant differences were detected among the remaining genotypes.

LMA did not differ significantly among genotypes under either treatment (Figure 5d,i).

Consistent with the growth responses, Fv/Fm and Pm were significantly lower in the *pgr5‐5* mutant than in the other genotypes under both FL600_10 and CL600_10 (Figure 5e,f,j,k).

When the *pgr5‐5* mutant was excluded from the analyses, no significant relationships between PGR5 abundance and dry weight, leaf area, Fv/Fm, or Pm were detected (Figure S21).

Together, these results indicate that the absence of PGR5 had a strong negative effect on plant growth under FL600_10 and CL600_10, whereas evidence for quantitative effects of PGR5 abundance among the overexpression lines was limited.

### Time‐course analysis of photosynthetic induction

To further characterize the effects of PGR5 abundance on photosynthetic induction, we analyzed the first 5 min of both the first and second induction phases and quantified the time required for ETRII and ETRI to reach 50–90% of their maximum values (t50–t90; Figures [Link], [Link], [Link]).

#### PSII induction

Under the 100 μmol photons m^−2^ s^−1^ induction protocol, ETRII time courses were similar among genotypes during both induction phases (Figure S10A,B). Nevertheless, the two highest PGR5‐expressing lines (PGR5ox 5‐10 and PGR5ox 25‐10) exhibited longer t50–t90 values than the other genotypes, indicating a slower induction of PSII electron transport (Figure S11A,B).

#### PSI induction

In contrast, PSI induction showed a clearer dependence on PGR5. The *pgr5‐5* mutant consistently exhibited lower ETRI and slower induction kinetics than the other genotypes during both induction phases (Figures S10C,D and S11C,D).

Under the 600 μmol photons m^−2^ s^−1^ induction protocol, the effects of PGR5 deficiency became more pronounced. The *pgr5‐5* mutant exhibited the lowest ETRII and ETRI values together with the slowest induction kinetics throughout the induction period (Figures S10E–H and S12A–D).

Overall, these analyses indicate that PGR5 contributes to efficient photosynthetic induction, particularly by maintaining rapid PSI electron transport during FL conditions.

## DISCUSSION

This study examined how different levels of PGR5 protein abundance affect photosynthetic induction, PSI redox regulation, and plant growth in *Arabidopsis thaliana*. Using a series of lines with graded PGR5 accumulation, we analyzed chlorophyll fluorescence parameters, P700 redox parameters, electron transport rates, and growth responses under defined dynamic light regimes.

A major finding of this study is that PGR5 abundance was associated with distinct photosynthetic responses during FL conditions. Under the 100 μmol photons m^−2^ s^−1^ induction protocol, PSI‐related parameters showed clearer associations with PGR5 abundance than PSII‐related parameters. In particular, Y(I), Y(ND), Y(NA), and ETRI differed among lines with different PGR5 abundance, whereas several PSII‐related responses were more strongly influenced by the presence or absence of PGR5 (Figure 2). Under the 600 μmol photons m^−2^ s^−1^ induction protocol, the *pgr5‐5* mutant showed strong impairment in both PSI‐ and PSII‐related parameters, indicating that the absence of PGR5 had a greater effect than quantitative variation in PGR5 abundance among the overexpression lines (Figure 3).

The growth analysis further showed that PGR5ox 65, which accumulated approximately twice the WT level of PGR5, exhibited the highest dry weight under FL100_10 conditions. However, this effect was most clearly observed in this single moderate overexpression line, and the remaining lines showed more limited differences. Therefore, our results support the idea that moderate PGR5 accumulation can be beneficial under FL100_10 conditions, but the evidence for a broad dose‐dependent relationship between PGR5 abundance and plant growth should be interpreted cautiously.

These findings extend previous studies demonstrating the importance of PGR5‐dependent photosynthetic regulation under FL conditions (Yamori & Shikanai, 2016). However, because CET activity was not directly measured in this study, we do not equate PGR5 abundance with CET activity. Instead, the observed phenotypes should be interpreted as PGR5‐dependent photosynthetic responses that are consistent with altered regulation of PSI‐associated electron transport. The molecular mechanism by which changes in PGR5 abundance influence photosynthetic regulation remains unresolved.

CET involves two major pathways: the PGR5‐dependent pathway and the chloroplast NDH‐dependent pathway (Yamori & Shikanai, 2016). Previous studies have demonstrated that the NDH‐dependent pathway also contributes to photosynthetic regulation, particularly under low light or moderate stress conditions (Yamori et al., 2011; Yamori et al., 2015). In the present study, we focused on the physiological effects of graded PGR5 protein abundance, whereas the contribution of the NDH pathway under the light regimes used here was not directly investigated. Therefore, further studies will be required to distinguish the respective roles of PGR5‐ and NDH‐dependent pathways in photosynthetic induction and photoprotection under dynamic light environments.

### Differences in fluctuating light regimes explain the non‐lethal phenotype of *pgr5‐5* in this study

Although the *pgr5‐5* mutant exhibited clear physiological impairment under the fluctuating light regimes used in this study, the plants remained viable throughout the growth period. Under FL600_10, the mutant showed significantly lower dry weight, leaf area, Fv/Fm, and Pm than WT and the PGR5 overexpression lines (Figure 5b,c,e,f).

This phenotype differs from previous reports in which *pgr5* mutants became lethal under fluctuating light (Suorsa et al., 2012; Tikkanen et al., 2010). We suggest that this difference primarily reflects the characteristics of the FL regimes used.

Previous studies generally employed frequent transitions between low and high irradiance, imposing severe stress on PSI (Kono et al., 2014; Suorsa et al., 2012). In contrast, our FL600_10 regime consisted of 10‐min periods at 600 μmol photons m^−2^ s^−1^ alternating with 10 μmol photons m^−2^ s^−1^, allowing sufficient time for photosynthetic induction while avoiding extremely rapid light fluctuations.

These differences in light regime likely reduced the severity of PSI stress, allowing the *pgr5‐5* mutant to survive despite its impaired photosynthetic performance. Our results therefore indicate that the severity of the pgr5 phenotype depends strongly on the temporal characteristics of the fluctuating light environment, rather than FL per se.

### Moderate PGR5 accumulation is associated with improved photosynthetic performance under FL100_10 conditions

The present study showed that the line with moderate PGR5 overexpression (PGR5ox 65) exhibited the greatest biomass accumulation under FL100_10 conditions. This line also showed distinct photosynthetic characteristics, including higher Y(I), lower Y(NA), and faster photosynthetic induction than the WT and the highest‐expressing lines. Together, these observations suggest that moderate PGR5 accumulation can be advantageous under this FL regime.

The beneficial effects of moderate PGR5 accumulation are consistent with the proposed role of PGR5 in regulating PSI redox balance during transitions from low to higher irradiance. During photosynthetic induction, downstream carbon metabolism requires time to become fully activated, increasing the risk of PSI over‐reduction. Previous studies have demonstrated that PGR5‐dependent regulation contributes to maintaining PSI oxidation through photosynthetic control at the cyt *b*_6_ *f* and by regulating electron flow on the acceptor side of PSI (Kono et al., 2025; Suorsa et al., 2012; Yamamoto & Shikanai, 2019; Zhou et al., 2022). The physiological responses observed in the present study are consistent with these proposed functions of PGR5.

At the same time, our results do not provide evidence that increasing PGR5 abundance per se proportionally enhances photosynthetic electron transport capacity. Several physiological responses were strongly influenced by the absence of PGR5, whereas relationships among the overexpression lines were generally weaker. Moreover, the increase in biomass under FL100_10 was most evident in the PGR5ox 65 line, while the remaining overexpression lines showed more limited differences.

Therefore, rather than supporting a simple dose‐dependent model, our results suggest that a moderate increase in PGR5 abundance may provide physiological benefits under specific conditions. Additional independent lines with intermediate PGR5 expression will be valuable for determining the generality of this relationship.

### Excessive PGR5 accumulation is associated with altered photosynthetic regulation

Although the PGR5 overexpression lines exhibited higher PGR5 protein abundance than WT, the highest‐expressing lines (PGR5ox 5‐10 and PGR5ox 25‐10) did not show further improvements in photosynthetic performance or plant growth. Instead, these lines exhibited lower Fv/Fm (Figure 1d), slower induction of Y(II) (Figure 2d), and higher NPQ during photosynthetic induction (Figure 2e).

In addition, transient changes in NPQ, Y(ND), and Y(NA) were observed during the induction response (Figures S2 and S3), indicating that photosynthetic regulation differed from that of WT and the moderately overexpressing line. These observations suggest that excessive accumulation of PGR5 alters the regulation of photosynthetic electron transport during repeated light transitions.

Previous studies have shown that PGR5 overexpression delays activation of the Calvin–Benson cycle (Okegawa et al., 2022). Although the present results are consistent with altered photosynthetic regulation in the highest‐expressing lines, the underlying mechanism remains unclear because neither CET activity nor Calvin–Benson cycle activation was directly examined in this study.

Taken together, our results suggest that both insufficient and excessive PGR5 accumulation are associated with suboptimal photosynthetic responses, whereas moderate PGR5 accumulation was associated with the most favorable physiological performance under the FL100_10 conditions examined here.

### Implications for photosynthesis under fluctuating light

Collectively, the present study demonstrates that PGR5 abundance influences photosynthetic regulation under dynamic light environments. In WT plants, PGR5 abundance is thought to be regulated by PGRL1 and PGRL2, which prevent excessive accumulation or degradation of PGR5 (Rühle et al., 2021). Our results suggest that moderate PGR5 accumulation was associated with improved photosynthetic performance and greater biomass accumulation under the FL100_10 conditions used in this study, whereas both the absence of PGR5 and excessive PGR5 accumulation were associated with less favorable physiological responses.

These findings highlight the importance of maintaining an appropriate level of PGR5 for efficient growth and photosynthetic electron transport during repeated changes in irradiance. Because natural light environments are highly dynamic, particularly within plant canopies, optimizing PGR5 abundance may represent one approach to improving photosynthetic performance under FL conditions. Further studies will be required to determine whether this relationship is conserved in crop species and under field conditions.

## CONCLUSION

This study demonstrates that altering PGR5 abundance influences photosynthetic regulation and plant performance under FL conditions. Moderate PGR5 accumulation was associated with improved photosynthetic induction, favorable PSI redox regulation, and greater biomass accumulation under the FL100_10 regime, whereas the absence of PGR5 caused severe impairment of photosynthetic performance, particularly under the FL600_10 regime. In contrast, excessively high PGR5 accumulation did not provide additional physiological benefits.

Overall, our findings suggest that maintaining an appropriate level of PGR5, rather than simply increasing its abundance, is important for efficient photosynthetic regulation under dynamic light environments. These results provide new physiological insights into the role of PGR5 in FL acclimation and offer a foundation for future studies investigating the optimization of photosynthesis in changing light environments.

## MATERIALS AND METHODS

### Plant materials and cultivation

We grew *Arabidopsis thaliana* wild type (ecotype Columbia *gl1*, WT), *PGR5* knockout mutant (*pgr5‐5*), and four transgenic lines overexpressing *PGR5* under the control of the cauliflower mosaic virus 35S promoter (*35S::PGR5*). The line names are *PGR5ox 20*, *PGR5ox 65*, *PGR5ox 5‐10*, and *PGR5ox 25‐10*, which differ in their PGR5 protein abundance. The knockout mutant was generated by Kobayashi et al. (2024), and these overexpression lines were generated by Okegawa et al. (2007) (Figure 1a).

All plants were grown in 400 mL pots or cell trays containing a 1:1 mixture of soil (Metro‐Mix 350J; Hyponex Japan, Osaka, Japan) and expanded vermiculite. They were cultivated under growth chamber conditions (Figure S13). Growth conditions were maintained at a temperature of 23°C, a relative humidity of 60%, and a 10‐h light/14‐h dark cycle with white light at 120 μmol photons m^−2^ s^−1^. The plants in pots were cultivated for 6 weeks, and Hyponex liquid fertilizer (Hyponex Japan, Osaka, Japan) was applied in week 4. These plants were then subjected to the photosynthetic induction measurements described below. Because the *pgr5‐5* mutant was grown separately from the other genotypes, all comparisons involving *pgr5‐5* were performed using plants cultivated under identical conditions within the same experiment.

For the growth experiments, five genotypes (WT, *pgr5‐5*, PGR5ox 65, PGR5ox 5‐10, and PGR5ox 25‐10) were grown in cell trays under continuous white light for 3 weeks before transfer to the light‐treatment systems. Four light regimes were used throughout this study: FL100_10, CL100_10, FL600_10, and CL600_10. In the FL treatments, plants were exposed to alternating 10‐min periods of 10 and either 100 or 600 μmol photons m^−2^ s^−1^. In the corresponding CL treatments, plants received the same total duration at each irradiance without fluctuations (2.5 h at 10 μmol photons m^−2^ s^−1^, followed by 5 h at either 100 or 600 μmol photons m^−2^ s^−1^, and then 2.5 h at 10 μmol photons m^−2^ s^−1^). Thus, each FL treatment and its corresponding CL treatment had identical daily light integrals and average irradiance but differed only in the temporal pattern of light exposure. The average irradiance was 55 μmol photons m^−2^ s^−1^ for FL100_10 and CL100_10, and 305 μmol photons m^−2^ s^−1^ for FL600_10 and CL600_10. These abbreviations are used consistently throughout the manuscript. Two white LED light sources (Figure S13B) were installed in a temperature‐controlled room maintained at 23°C, with one assigned to the FL treatment and the other to the CL treatment. All treatments were conducted under a 10‐h light/14‐h dark photoperiod. Two independent cell trays were placed in each light‐treatment system for 3 weeks before chlorophyll fluorescence and P700 measurements were performed. The growth experiments were conducted independently at least twice, and similar trends were consistently observed across experiments.

### Measurement of chlorophyll fluorescence and P700 in a photosynthetic induction response

Chlorophyll fluorescence and P700 were measured simultaneously with a Dual‐PAM‐100 measuring system (Heinz Walz GmbH, Effeltrich, Germany), as described previously (Kono et al., 2025; Qu et al., 2025). Measurements were performed on fully expanded, uppermost leaves of 6‐week‐old plants. Leaves were dark‐adapted for a day, and then, a saturating pulse was applied to obtain F_v_/F_m_ (Genty et al., 1989) and Pm (Klughammer & Schreiber, 1994; Klughammer & Schreiber, 2008). Chlorophyll fluorescence was determined with a Dual‐PAM‐100's chamber. First, a saturating pulse was applied to plant leaves to obtain maximum fluorescence. After measuring of Y(II), we calculated ETRII as:

(1) $$\text{ETRII}=0.5\times I_{\mathrm{abs}}\times Y\left(\mathrm{II}\right)$$

The P700 was also determined with the Dual‐PAM‐100's chamber. First, leaves were treated with far‐red light and a saturating pulse to obtain P_m_. After measurement of Y(I), we calculated ETRI as follows:

(2) $$\begin{array}{c} \text{ETRI}=0.5\times I_{\mathrm{abs}}\times Y\left(I\right) \end{array}$$

where 0.5 is the fraction of absorbed light allocated to each photosystem, and *I*_*abs*_ is the absorbed irradiance, taken as 0.84 of the incident irradiance. In addition, NPQ (Bilger & Björkman, 1990), 1 − qL (Kramer et al., 2004), Y(ND) (Klughammer & Schreiber, 2008), and Y(NA) (Klughammer & Schreiber, 2008) were calculated. NPQ represents the extent of non‐photochemical quenching, whereas 1 − qL reflects the redox state of the plastoquinone pool, specifically the primary electron acceptor Q_A_. Y(ND) is the quantum yield of non‐photochemical energy dissipation due to donor side limitation, and Y(NA) is the quantum yield of non‐photochemical energy dissipation due to acceptor‐side limitation.

Chlorophyll fluorescence and P700 were measured every 60 sec at a CO_2_ concentration of 400 μmol mol^−1^, 60% RH, and 23.0°C.

Two photosynthetic induction protocols were used throughout this study. In the 600 μmol photons m^−2^ s^−1^ induction protocol, actinic light was applied sequentially as follows: darkness (3 min), 10 μmol photons m^−2^ s^−1^ (5 min), 600 μmol photons m^−2^ s^−1^ (30 min), 10 μmol photons m^−2^ s^−1^ (5 min), 600 μmol photons m^−2^ s^−1^ (10 min), and finally darkness (5 min). In the 100 μmol photons m^−2^ s^−1^ induction protocol (Figure 3a), the same temporal sequence was used except that the peak irradiance was 100 μmol photons m^−2^ s^−1^ instead of 600 μmol photons m^−2^ s^−1^ (Figure 2a). For subsequent analyses, the first exposure to either 100 or 600 μmol photons m^−2^ s^−1^ was defined as the first induction, the subsequent constant‐light period as the steady‐state phase, and the second exposure as the second induction (Figures 2a, 3a). These terms are used consistently throughout the manuscript.

For the investigation of photosynthetic induction, plants were selected at random and measured from 08:00 to 16:00 to avoid confounding samples with the time of day and to minimize any diurnal influences. The day before measurements, plants were removed from the growth chamber before the light period began and were kept in a dark room at an air temperature of 25°C, relative humidity of 65% and ambient CO_2_ concentration. A leaf was then placed into the measuring chamber while maintaining dark conditions, with an air temperature of 23°C, relative humidity of 60% and 400 μmol CO_2_ mol^−1^.

To quantify induction kinetics, ETRII and ETRI time courses following each increase in irradiance were fitted with quadratic functions, and t50–t90 values were calculated from the fitted curves.

### Measurement of chlorophyll fluorescence, P700 and growth analysis under a long‐term fluctuating light condition

Chlorophyll fluorescence and P_700_ were also measured simultaneously with a Dual‐PAM‐100 measuring system. Plants grown under the four long‐term light regimes (FL100_10, CL100_10, FL600_10, and CL600_10) were measured immediately after the 3‐week treatment. All measurements were performed under controlled environmental conditions maintained at 25°C, 65 %RH, and ambient CO_2_ concentration. Leaves were dark‐adapted before measurement, after which Fv/Fm was determined, followed by measurement of Pm.

Following these measurements, the plants were subjected to growth analysis. The entire shoot of each plant was first scanned at 600 dpi using a flatbed scanner (CanoScan LiDE 220, Canon Inc., Tokyo, Japan) to create a digital image. The total leaf area was subsequently calculated from these images using ImageJ software (National Institutes of Health, MD, USA). After scanning, each plant was placed into an individual paper envelope and dried in an oven at 80°C for 3 days. The total dry mass was then determined by weighing each dried sample. Finally, the LMA was calculated by dividing the total dry mass by the leaf area.

### Statistical analysis

Differences among three or more groups were analyzed by ANOVA using the Tukey–Kramer test according to Sakoda et al. (2021). Throughout the manuscript, statistical comparisons among genotypes are based primarily on the results of the Tukey–Kramer test. Pearson's correlation coefficient (r) was calculated, and the significance of relationships was tested by two‐sided *t*‐tests (*P* < 0.05). Regression analyses were used to visualize relationships between PGR5 abundance and physiological traits and were interpreted together with the multiple‐comparison results. All statistical analyses were conducted in Python 3.13.4 (Python Software Foundation, DE, USA).

### Western blotting of PGR5 and PetC proteins and SDS‐PAGE of Rubisco

Leaf tissues were sampled after chlorophyll fluorescence and P700 measurements, frozen in liquid nitrogen and stored at −80°C until analysis. The Rubisco content was determined by formamide extraction of SDS‐PAGE separated, Coomassie Brilliant Blue R‐250 stained bands corresponding to the large and small subunits of Rubisco as described in Qu et al. (2021). The proteins were separated by 16.5% SDS‐PAGE using the conventional Tris‐tricine buffer system for PGR5 and PetC proteins (Schägger & von Jagow, 1987) and transferred onto a PVDF (polyvinylidene fluoride) membrane. Immunodetection with antibodies against PGR5 and PetC was performed as described in Munekage et al. (2002) and Okegawa et al. (2007). For immunoblot analysis, the loading volume of each sample was adjusted to normalize chlorophyll content across all lanes. Extracts from four WT individuals were pooled based on their chlorophyll content to create a ‘WT mix’, which was used as a protein loading control.

## AUTHOR CONTRIBUTIONS

KT and WY conceived and designed the study. KT, HK, YO, and WY conducted experiments and performed data analysis. KT, YO, TS, and WY wrote the manuscript. All authors discussed the results and revised the manuscript.

## CONFLICT OF INTEREST

The authors declare no conflict of interest.

## AVAILABILITY OF MATERIALS

The materials generated in this study are available from the corresponding author upon reasonable request.

## Supporting information


**Figure S1.** Protein accumulation in WT, *pgr5‐5*, *PGR5ox 20*, *PGR5ox 65*, *PGR5ox 5‐10* and *PGR5ox 25‐10*. (A) Relative PetC protein abundance, with WT set to 100%. (B) Relative Rubisco large subunit (RbcL) abundance, with WT set to 100%. Sample loading was normalized on a chlorophyll basis. Data are means ± SE (n ≥ 3). Individual data points are shown. Different letters indicate significant differences among genotypes (one‐way ANOVA followed by Tukey's HSD test, *P* < 0.05).

**Figure S2.** Chlorophyll fluorescence and P700 analysis of a photosynthetic induction response in WT, *pgr5‐5*, *PGR5ox 20*, *PGR5ox 65*, *PGR5ox 5‐10* and *PGR5ox 25‐10* under the 100 μmol photons m^−2^ s^−1^ induction protocol. (A) NPQ, (B) 1 − qL, (C) Y(ND), (D) Y(NA), (E) ETRII, and (F) ETRI. Data are means ± SE (n ≥ 4).

**Figure S3.** Enlarged views of the initial photosynthetic induction phase under the 100 μmol photons m^−2^ s^−1^ induction protocol. (A) NPQ, (B) Y(ND), and (C) Y(NA) immediately following the transition from low to higher irradiance. Data are means ± SE (n ≥ 4).

**Figure S4.** Chlorophyll fluorescence and P700 analysis of a photosynthetic induction response in WT, *pgr5‐5*, *PGR5ox 20*, *PGR5ox 65*, *PGR5ox 5‐10* and *PGR5ox 25‐10* under the 100 μmol photons m^−2^ s^−1^ induction protocol. (A–F) Relationships between PGR5 protein abundance and Y(II), NPQ, 1 − qL, Y(I), Y(ND), and Y(NA), respectively. Data are means ± SE (n ≥ 4). Different letters indicate significant differences among genotypes (one‐way ANOVA followed by Tukey's HSD test, *P* < 0.05). Regression lines were fitted using the least‐squares method.

**Figure S5.** Chlorophyll fluorescence and P700 analysis of a photosynthetic induction response in WT, *pgr5‐5*, *PGR5ox 20*, *PGR5ox 65*, *PGR5ox 5‐10* and *PGR5ox 25‐10* under the 100 μmol photons m^−2^ s^−1^ induction protocol. (A, B) Relationships between PGR5 protein abundance and ETRII and ETRI, respectively, during the first induction phase. (C, D) Corresponding relationships during the steady‐state phase. (E, F) Corresponding relationships during the post transition phase. (G, H) Corresponding relationships during the second induction phase. Data are means ± SE (n ≥ 4). Different letters indicate significant differences among genotypes (one‐way ANOVA followed by Tukey's HSD test, *P* < 0.05). Regression lines were fitted using the least‐squares method.

**Figure S6.** Chlorophyll fluorescence and P700 analysis of a photosynthetic induction response in WT, *pgr5‐5*, *PGR5ox 20*, *PGR5ox 65*, *PGR5ox 5‐10* and *PGR5ox 25‐10* under the 600 μmol photons m^−2^ s^−1^ induction protocol. (A) NPQ, (B) 1 − qL, (C) Y(ND), (D) Y(NA), (E) ETRII, and (F) ETRI. Data are means ± SE (n ≥ 4).

**Figure S7.** Chlorophyll fluorescence and P700 analysis of a photosynthetic induction response in WT, *pgr5‐5*, *PGR5ox 20*, *PGR5ox 65*, *PGR5ox 5‐10* and *PGR5ox 25‐10* under the 600 μmol photons m^−2^ s^−1^ induction protocol. (A–F) Relationships between PGR5 protein abundance and Y(II), NPQ, 1 − qL, Y(I), Y(ND), and Y(NA), respectively. Data are means ± SE (n ≥ 4). Different letters indicate significant differences among genotypes (one‐way ANOVA followed by Tukey's HSD test, *P* < 0.05). Regression lines were fitted using the least‐squares method.

**Figure S8.** Chlorophyll fluorescence and P700 analysis of a photosynthetic induction response in WT, *pgr5‐5*, *PGR5ox 20*, *PGR5ox 65*, *PGR5ox 5‐10* and *PGR5ox 25‐10* under the 600 μmol photons m^−2^ s^−1^ induction protocol. (A, B) Relationships between PGR5 protein abundance and ETRII and ETRI, respectively, during the first induction phase. (C, D) Corresponding relationships during the steady‐state phase. (E, F) Corresponding relationships during the post transition phase. (G, H) Corresponding relationships during the second induction phase. Data are means ± SE (n ≥ 4). Different letters indicate significant differences among genotypes (one‐way ANOVA followed by Tukey's HSD test, *P* < 0.05). Regression lines were fitted using the least‐squares method.

**Figure S9.** Chlorophyll fluorescence and P700 analysis of a photosynthetic induction response in WT, *pgr5‐5*, *PGR5ox 20*, *PGR5ox 65*, *PGR5ox 5‐10* and *PGR5ox 25‐10*. Relationships between PGR5 protein abundance and t50 of (A) ETRII under the 100 μmol photons m^−2^ s^−1^ induction protocol, (B) ETRI under the 100 μmol photons m^−2^ s^−1^ induction protocol, (C) ETRII under the 600 μmol photons m^−2^ s^−1^ induction protocol, and (D) ETRI under the 600 μmol photons m^−2^ s^−1^ induction protocol during the first induction phase. Data are means ± SE (n ≥ 4). Different letters indicate significant differences among genotypes (one‐way ANOVA followed by Tukey's HSD test, *P* < 0.05). Regression lines were fitted using the least‐squares method.

**Figure S10.** Time‐course changes in electron transport rates during the 100 and 600 μmol photons m^−2^ s^−1^ induction protocols in WT, *pgr5‐5*, PGR5ox 20, PGR5ox 65, PGR5ox 5‐10 and PGR5ox 25‐10. (A, B) ETRII during the first and second induction phases under the 100 μmol photons m^−2^ s^−1^ induction protocol. (C, D) ETRI during the first and second induction phases under the 100 μmol photons m^−2^ s^−1^ induction protocol. (E, F) ETRII during the first and second induction phases under the 600 μmol photons m^−2^ s^−1^ induction protocol. (G, H) ETRI during the first and second induction phases under the 600 μmol photons m^−2^ s^−1^ induction protocol. Data are means ± SE (n ≥ 4).

**Figure S11.** Induction kinetics under the 100 μmol photons m^−2^ s^−1^ induction protocol. Relationships between PGR5 protein abundance and the time required for ETRII and ETRI to increase from 50% to 90% of their maximum values (t50–t90). (A, B) ETRII during the first and second induction phases, respectively. (C, D) ETRI during the first and second induction phases, respectively. Data are means ± SE (n ≥ 4). Different letters indicate significant differences among genotypes (one‐way ANOVA followed by Tukey's HSD test, *P* < 0.05). Regression lines were fitted using the least‐squares method.

**Figure S12.** Induction kinetics under the 600 μmol photons m^−2^ s^−1^ induction protocol. Relationships between PGR5 protein abundance and the time required for ETRII and ETRI to increase from 50% to 90% of their maximum values (t50–t90). (A, B) ETRII during the first and second induction phases, respectively. (C, D) ETRI during the first and second induction phases, respectively. Data are means ± SE (n ≥ 4). Different letters indicate significant differences among genotypes (one‐way ANOVA followed by Tukey's HSD test, *P* < 0.05). Regression lines were fitted using the least‐squares method.

**Figure S13.** Growth conditions used in this study. (A) Growth chamber used for six‐week cultivation prior to photosynthetic induction measurements. (B) Experimental system used for long‐term FL100_10, CL100_10, FL600_10, and CL600_10 treatments. Spectral photon flux density distributions of the white LED light sources used for plant cultivation.

**Figure S14.** Relationships between PGR5 protein abundance and photosynthetic parameters during the 100 μmol photons m^−2^ s^−1^ induction protocol after excluding the *pgr5‐5* mutant. (A–F) Relationships between PGR5 protein abundance and Y(II), NPQ, 1 − qL, Y(I), Y(ND), and Y(NA), respectively, during the first induction phase. (G–L) Corresponding relationships during the steady‐state phase. (M–R) Corresponding relationships during the post transition phase. Data are means ± SE (n ≥ 4). Regression lines were fitted using the least‐squares method. Pearson's correlation coefficient (r) and *P*‐values are shown in each panel.

**Figure S15.** Relationships between PGR5 protein abundance and electron transport rates during the 100 μmol photons m^−2^ s^−1^ induction protocol after excluding the *pgr5‐5* mutant. (A–C) Relationships between PGR5 protein abundance and ETRII during the first induction, steady‐state, and second induction phases, respectively. (D–F) Corresponding relationships for ETRI. Data are means ± SE (n ≥ 4). Regression lines were fitted using the least‐squares method. Pearson's correlation coefficient (r) and *P*‐values are shown in each panel.

**Figure S16.** Chlorophyll fluorescence and P700 analysis of a photosynthetic induction response under the 100 μmol photons m^−2^ s^−1^ induction protocol after excluding the *pgr5‐5* mutant. (A, B) Relationships between PGR5 protein abundance and ETRII and ETRI, respectively, during the first induction phase. (C, D) Corresponding relationships during the steady‐state phase. (E, F) Corresponding relationships during the post transition phase. (G, H) Corresponding relationships during the second induction phase. Data are means ± SE (n ≥ 4). Different letters indicate significant differences among genotypes (one‐way ANOVA followed by Tukey's HSD test, *P* < 0.05). Regression lines were fitted using the least‐squares method.

**Figure S17.** Relationships between PGR5 protein abundance and photosynthetic parameters during the 600 μmol photons m^−2^ s^−1^ induction protocol after excluding the *pgr5‐5* mutant. (A–F) Relationships between PGR5 protein abundance and Y(II), NPQ, 1 − qL, Y(I), Y(ND), and Y(NA), respectively, during the first induction phase. (G–L) Corresponding relationships during the steady‐state phase. (M–R) Corresponding relationships during the post transition phase. Data are means ± SE (n ≥ 4). Regression lines were fitted using the least‐squares method. Pearson's correlation coefficient (r) and *P*‐values are shown in each panel.

**Figure S18.** Relationships between PGR5 protein abundance and electron transport rates during the 600 μmol photons m^−2^ s^−1^ induction protocol after excluding the *pgr5‐5* mutant. (A–C) Relationships between PGR5 protein abundance and ETRII during the first induction, steady‐state, and second induction phases, respectively. (D–F) Corresponding relationships for ETRI. Data are means ± SE (n ≥ 4). Regression lines were fitted using the least‐squares method. Pearson's correlation coefficient (r) and *P*‐values are shown in each panel.

**Figure S19.** Chlorophyll fluorescence and P700 analysis of a photosynthetic induction response under the 600 μmol photons m^−2^ s^−1^ induction protocol after excluding the *pgr5‐5* mutant. (A, B) Relationships between PGR5 protein abundance and ETRII and ETRI, respectively, during the first induction phase. (C, D) Corresponding relationships during the steady‐state phase. (E, F) Corresponding relationships during the post transition phase. (G, H) Corresponding relationships during the second induction phase. Data are means ± SE (n ≥ 4). Different letters indicate significant differences among genotypes (one‐way ANOVA followed by Tukey's HSD test, *P* < 0.05). Regression lines were fitted using the least‐squares method.

**Figure S20.** Relationships between PGR5 protein abundance and growth traits after excluding the *pgr5‐5* mutant under FL100_10 and CL100_10 conditions. (A–E) Relationships between PGR5 protein abundance and shoot dry weight, leaf area, LMA, Fv/Fm, and Pm, respectively, under FL100_10. (F–J) Corresponding relationships under CL100_10. Data are means ± SE (n = 4). Regression lines were fitted using the least‐squares method. Pearson's correlation coefficient (r) and *P*‐values are shown in each panel.

**Figure S21.** Relationships between PGR5 protein abundance and growth traits after excluding the *pgr5‐5* mutant under FL600_10 and CL600_10 conditions. (A–E) Relationships between PGR5 protein abundance and shoot dry weight, leaf area, LMA, Fv/Fm, and Pm, respectively, under FL600_10. (F–J) Corresponding relationships under CL600_10. Data are means ± SE (n = 4). Regression lines were fitted using the least‐squares method. Pearson's correlation coefficient (r) and *P*‐values are shown in each panel.

## Data Availability

The data supporting this work are available within the paper and its Supporting Information.
