# Supplementary material for: Moderate overexpression of PROTON GRADIENT REGULATION 5 improves photosynthetic performance and plant growth under fluctuating low light in Arabidopsis thaliana
Source: Plant J. 2026 Sep 28;127(6):e71120. doi: 10.1111/tpj.71120 (PMC13620446; doi:10.1111/tpj.71120)
Supplement: Supplementary file 2 [file TPJ-127-0-s002.pdf]

Figure S15

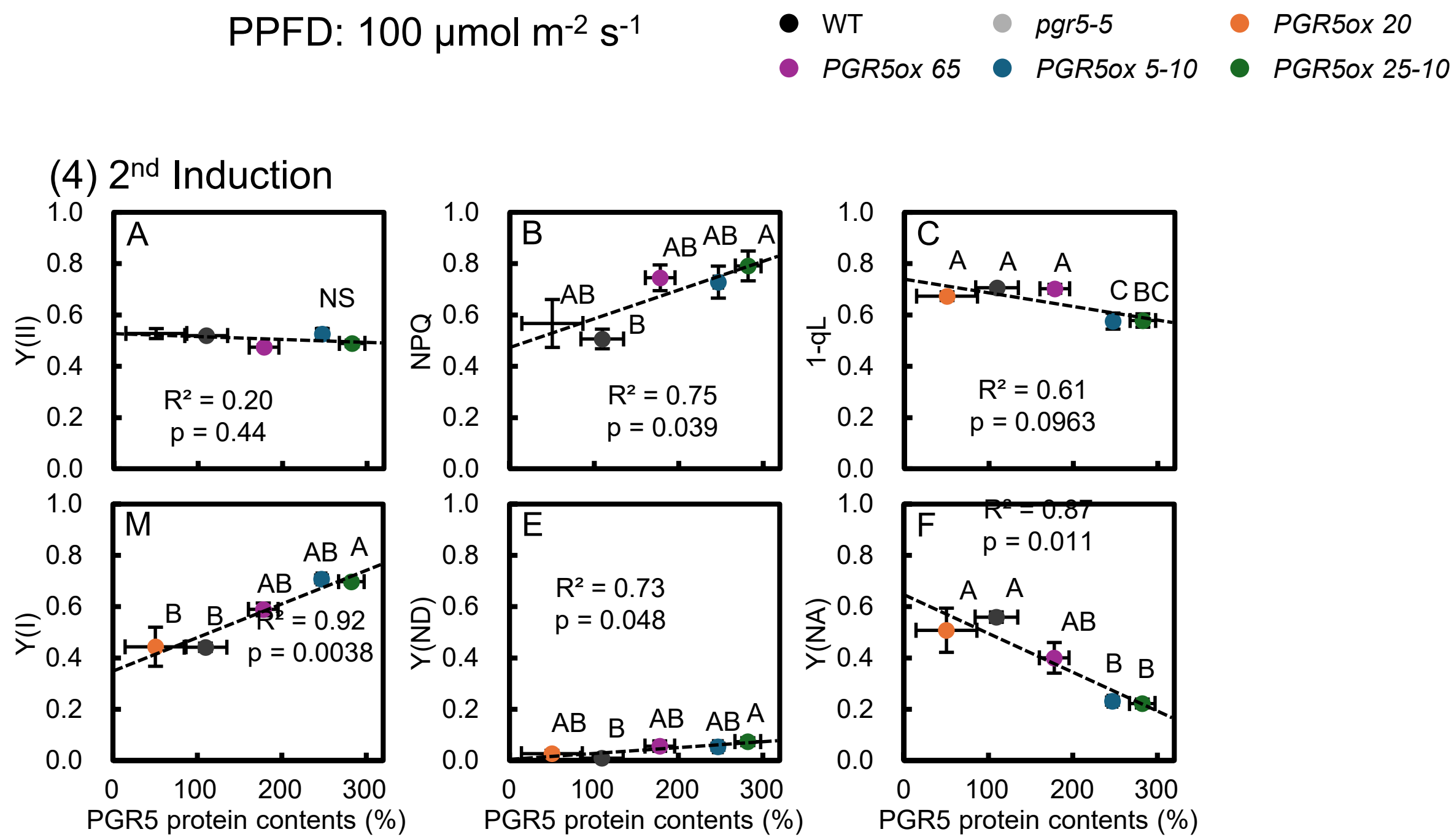

PPFD: 100 µmol m-2 s-1
WT
pgr5-5
PGR5ox 20
PGR5ox 65
PGR5ox 5-10
PGR5ox 25-10
(4) 2nd Induction
A
Y(II)
NS
R² = 0.20
p = 0.44
B
NPQ
AB
AB
AB
A
B
R² = 0.75
p = 0.039
C
1-qL
A
A
A
C
BC
R² = 0.61
p = 0.0963
M
Y(I)
B
B
AB
AB
A
R² = 0.92
p = 0.0038
E
Y(ND)
R² = 0.73
p = 0.048
AB
B
AB
AB
A
F
Y(NA)
R² = 0.87
p = 0.011
A
A
AB
B
B
PGR5 protein contents (%)
