# Supplementary material for: Moderate overexpression of PROTON GRADIENT REGULATION 5 improves photosynthetic performance and plant growth under fluctuating low light in Arabidopsis thaliana
Source: Plant J. 2026 Sep 28;127(6):e71120. doi: 10.1111/tpj.71120 (PMC13620446; doi:10.1111/tpj.71120)
Supplement: Supplementary file 3 [file TPJ-127-0-s003.pdf]

Figure S3

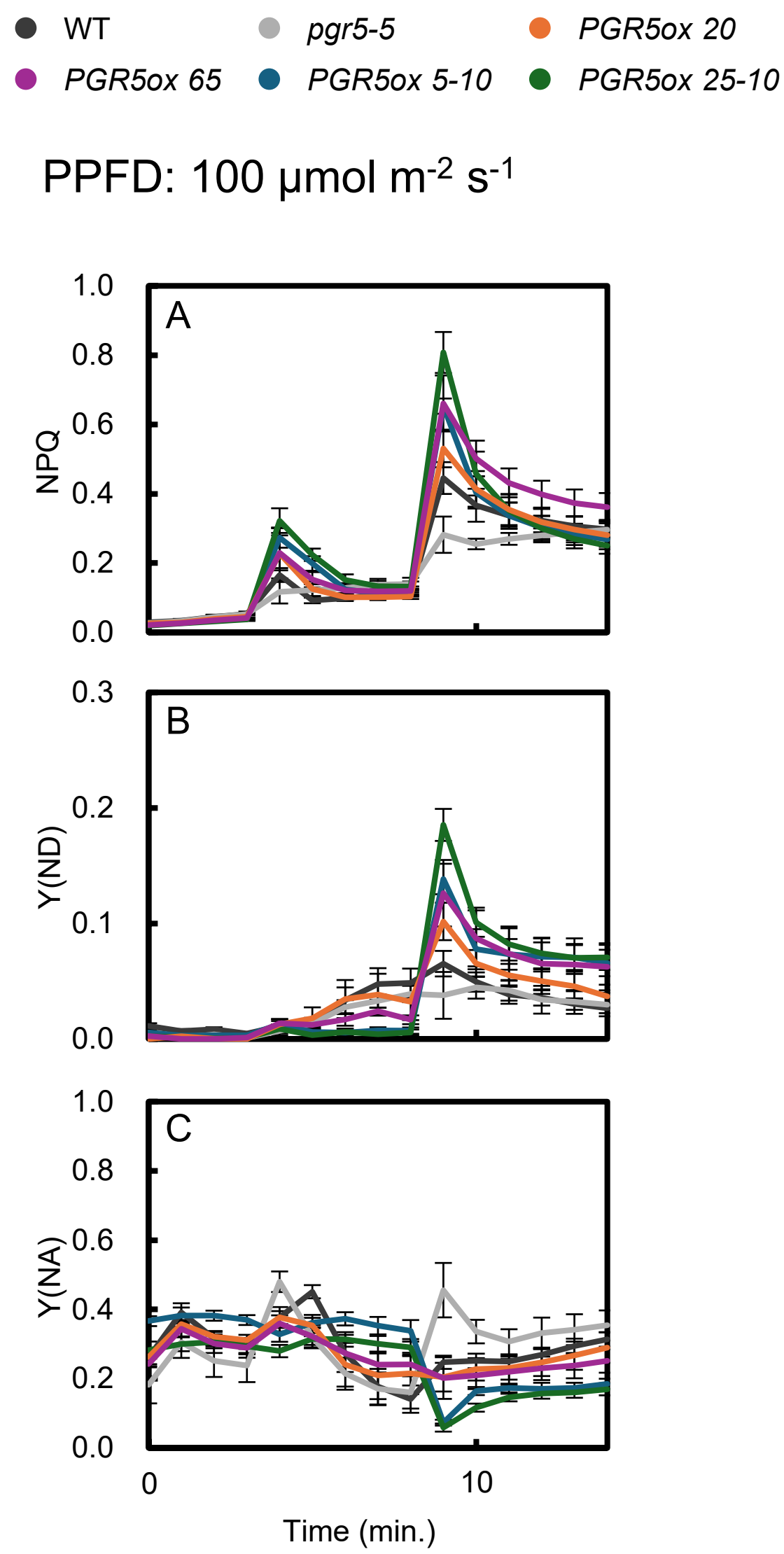

WT
pgr5-5
PGR5ox 20
PGR5ox 65
PGR5ox 5-10
PGR5ox 25-10
PPFD: 100 µmol m-2 s-1
A
B
C
NPQ
Y(ND)
Y(NA)
1.0
0.8
0.6
0.4
0.2
0.0
0.3
0.2
0.1
0.0
0
10
Time (min.)
