# Supplementary material for: Moderate overexpression of PROTON GRADIENT REGULATION 5 improves photosynthetic performance and plant growth under fluctuating low light in Arabidopsis thaliana
Source: Plant J. 2026 Sep 28;127(6):e71120. doi: 10.1111/tpj.71120 (PMC13620446; doi:10.1111/tpj.71120)
Supplement: Supplementary file 4 [file TPJ-127-0-s004.pdf]

Figure S16

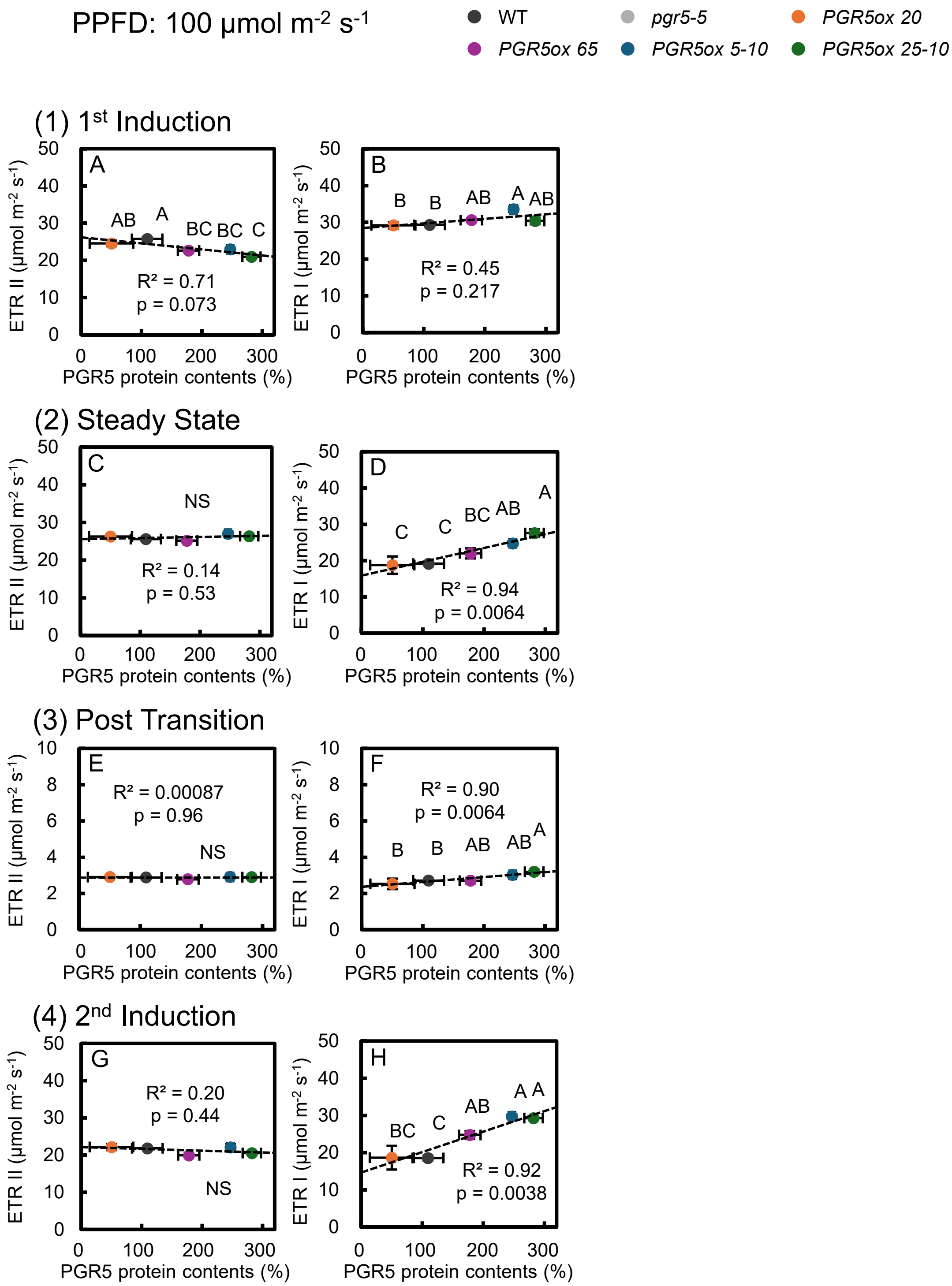
PPFD: 100 μmol m-2 s-1
WT
pgr5-5
PGR5ox 20
PGR5ox 65
PGR5ox 5-10
PGR5ox 25-10
(1) 1st Induction
A
AB
A
BC
BC
C
R² = 0.71
p = 0.073
B
B
B
AB
A
AB
R² = 0.45
p = 0.217
(2) Steady State
C
NS
R² = 0.14
p = 0.53
D
C
C
BC
AB
A
R² = 0.94
p = 0.0064
(3) Post Transition
E
R² = 0.00087
p = 0.96
NS
F
R² = 0.90
p = 0.0064
B
B
AB
AB
A
(4) 2nd Induction
G
R² = 0.20
p = 0.44
NS
H
BC
C
AB
A
A
R² = 0.92
p = 0.0038
ETR II (μmol m-2 s-1)
ETR I (μmol m-2 s-1)
PGR5 protein contents (%)
