# Supplementary material for: Moderate overexpression of PROTON GRADIENT REGULATION 5 improves photosynthetic performance and plant growth under fluctuating low light in Arabidopsis thaliana
Source: Plant J. 2026 Sep 28;127(6):e71120. doi: 10.1111/tpj.71120 (PMC13620446; doi:10.1111/tpj.71120)
Supplement: Supplementary file 5 [file TPJ-127-0-s005.pdf]

Figure S20

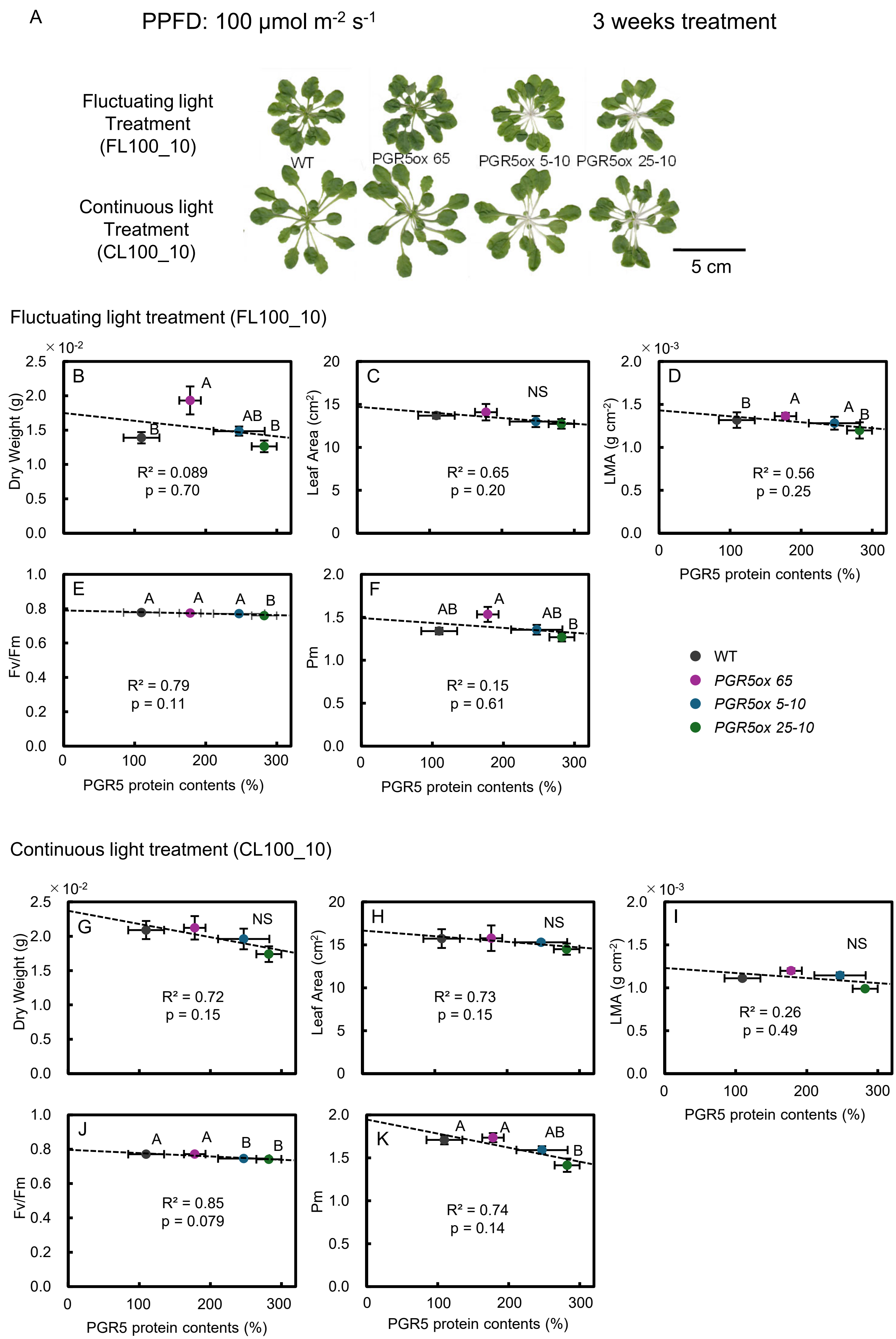
A
PPFD: 100 μmol m-2 s-1
3 weeks treatment
Fluctuating light Treatment (FL100_10)
Continuous light Treatment (CL100_10)
WT
PGR5ox 65
PGR5ox 5-10
PGR5ox 25-10
5 cm
Fluctuating light treatment (FL100_10)
B
Dry Weight (g)
× 10-2
R² = 0.089
p = 0.70
C
Leaf Area (cm²)
NS
R² = 0.65
p = 0.20
D
LMA (g cm-2)
× 10-3
R² = 0.56
p = 0.25
E
Fv/Fm
R² = 0.79
p = 0.11
F
Pm
R² = 0.15
p = 0.61
PGR5 protein contents (%)
WT
PGR5ox 65
PGR5ox 5-10
PGR5ox 25-10
Continuous light treatment (CL100_10)
G
NS
R² = 0.72
p = 0.15
H
NS
R² = 0.73
p = 0.15
I
NS
R² = 0.26
p = 0.49
J
R² = 0.85
p = 0.079
K
R² = 0.74
p = 0.14
PGR5 protein contents (%)
