# Supplementary material for: Moderate overexpression of PROTON GRADIENT REGULATION 5 improves photosynthetic performance and plant growth under fluctuating low light in Arabidopsis thaliana
Source: Plant J. 2026 Sep 28;127(6):e71120. doi: 10.1111/tpj.71120 (PMC13620446; doi:10.1111/tpj.71120)
Supplement: Supplementary file 6 [file TPJ-127-0-s006.pdf]

Figure S19

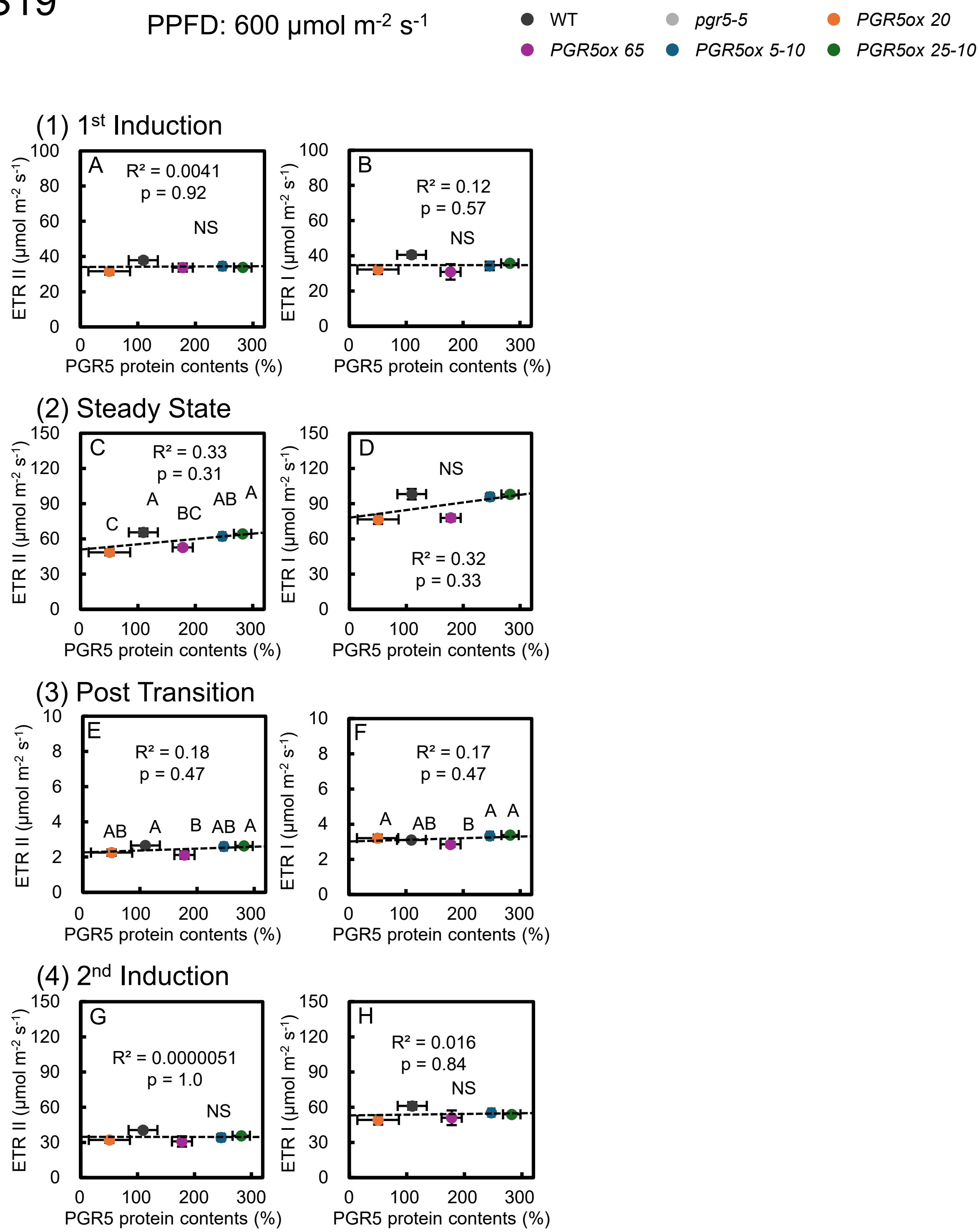
PPFD: 600 μmol m-2 s-1
WT
pgr5-5
PGR5ox 20
PGR5ox 65
PGR5ox 5-10
PGR5ox 25-10
(1) 1st Induction
A
R² = 0.0041
p = 0.92
NS
B
R² = 0.12
p = 0.57
NS
(2) Steady State
C
R² = 0.33
p = 0.31
C
A
BC
AB
A
D
NS
R² = 0.32
p = 0.33
(3) Post Transition
E
R² = 0.18
p = 0.47
AB
A
B
AB
A
F
R² = 0.17
p = 0.47
A
AB
B
A
A
(4) 2nd Induction
G
R² = 0.0000051
p = 1.0
NS
H
R² = 0.016
p = 0.84
NS
ETR II (μmol m-2 s-1)
ETR I (μmol m-2 s-1)
PGR5 protein contents (%)
