# Supplementary material for: Moderate overexpression of PROTON GRADIENT REGULATION 5 improves photosynthetic performance and plant growth under fluctuating low light in Arabidopsis thaliana
Source: Plant J. 2026 Sep 28;127(6):e71120. doi: 10.1111/tpj.71120 (PMC13620446; doi:10.1111/tpj.71120)
Supplement: Supplementary file 7 [file TPJ-127-0-s007.pdf]

Figure S7

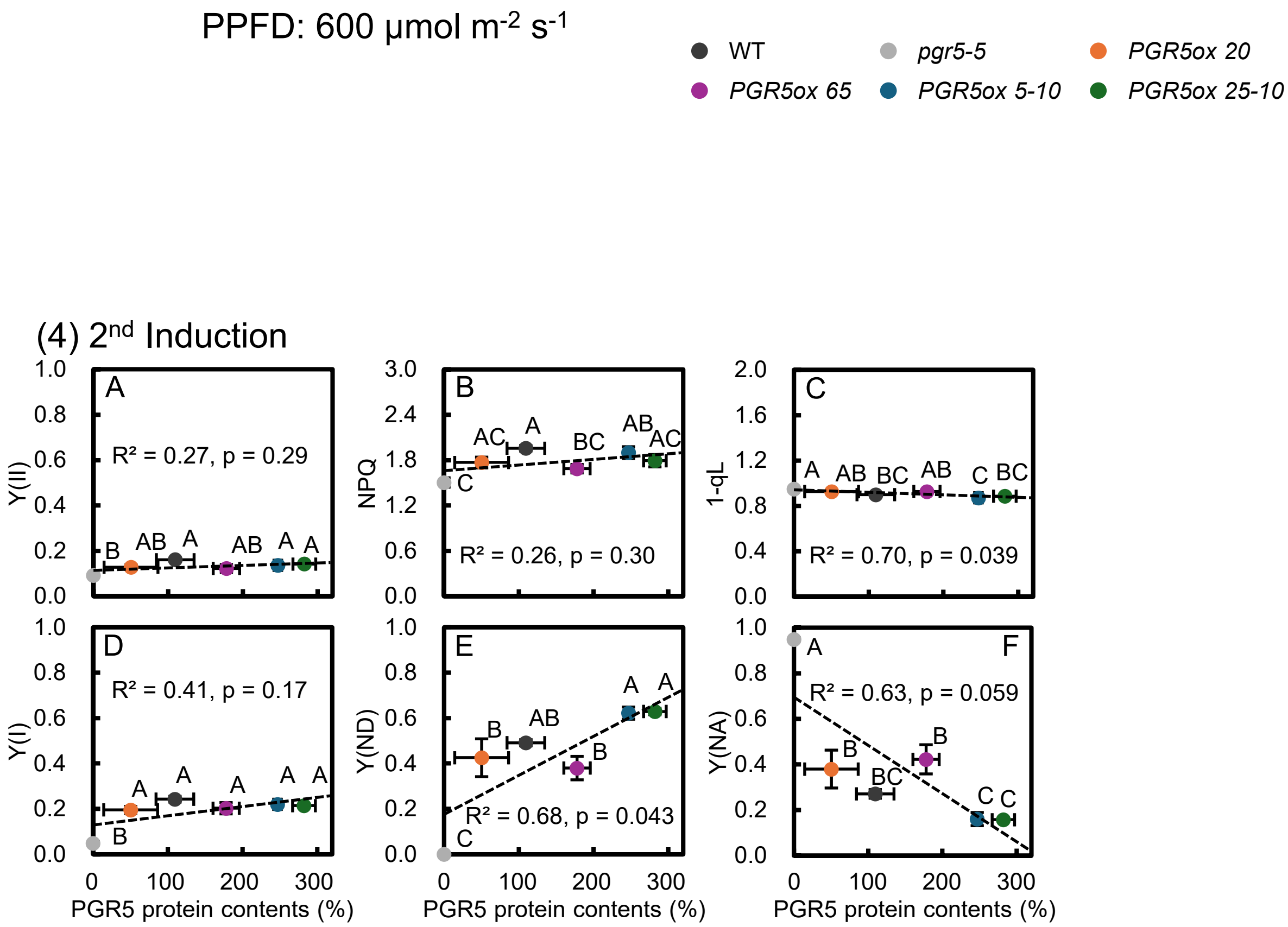
PPFD: 600 µmol m-2 s-1
WT
pgr5-5
PGR5ox 20
PGR5ox 65
PGR5ox 5-10
PGR5ox 25-10
(4) 2nd Induction
A
R² = 0.27, p = 0.29
Y(II)
B
R² = 0.26, p = 0.30
NPQ
C
R² = 0.70, p = 0.039
1-qL
D
R² = 0.41, p = 0.17
Y(I)
E
R² = 0.68, p = 0.043
Y(ND)
F
R² = 0.63, p = 0.059
Y(NA)
PGR5 protein contents (%)
