# Supplementary material for: Moderate overexpression of PROTON GRADIENT REGULATION 5 improves photosynthetic performance and plant growth under fluctuating low light in Arabidopsis thaliana
Source: Plant J. 2026 Sep 28;127(6):e71120. doi: 10.1111/tpj.71120 (PMC13620446; doi:10.1111/tpj.71120)
Supplement: Supplementary file 8 [file TPJ-127-0-s008.pdf]

Figure S12

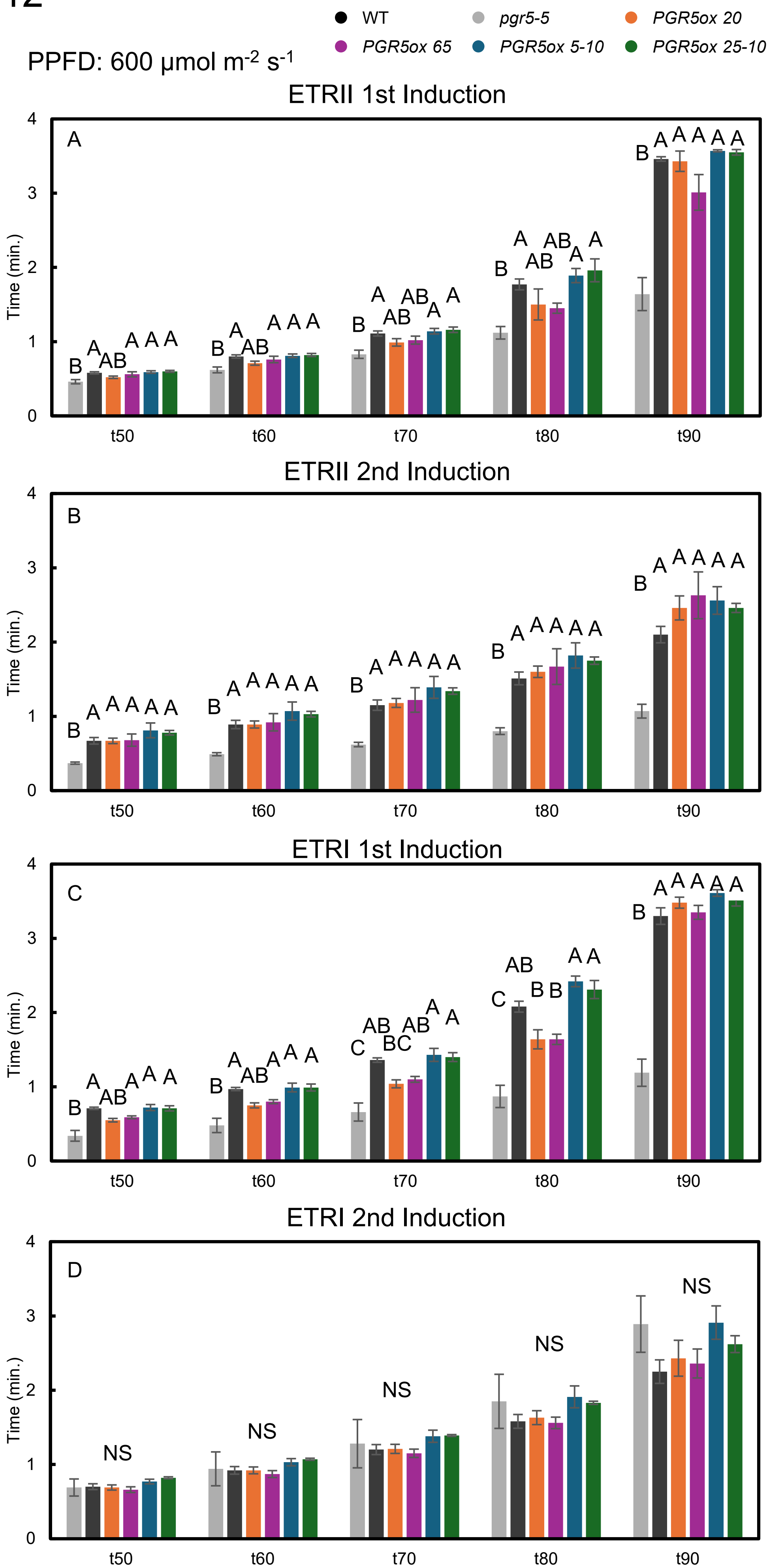
WT
pgr5-5
PGR5ox 20
PGR5ox 65
PGR5ox 5-10
PGR5ox 25-10
PPFD: 600 μmol m-2 s-1
ETRII 1st Induction
A
ETRII 2nd Induction
B
ETRI 1st Induction
C
ETRI 2nd Induction
D
Time (min.)
t50
t60
t70
t80
t90
NS
