# Supplementary material for: Moderate overexpression of PROTON GRADIENT REGULATION 5 improves photosynthetic performance and plant growth under fluctuating low light in Arabidopsis thaliana
Source: Plant J. 2026 Sep 28;127(6):e71120. doi: 10.1111/tpj.71120 (PMC13620446; doi:10.1111/tpj.71120)
Supplement: Supplementary file 9 [file TPJ-127-0-s009.pdf]

# Figure S5

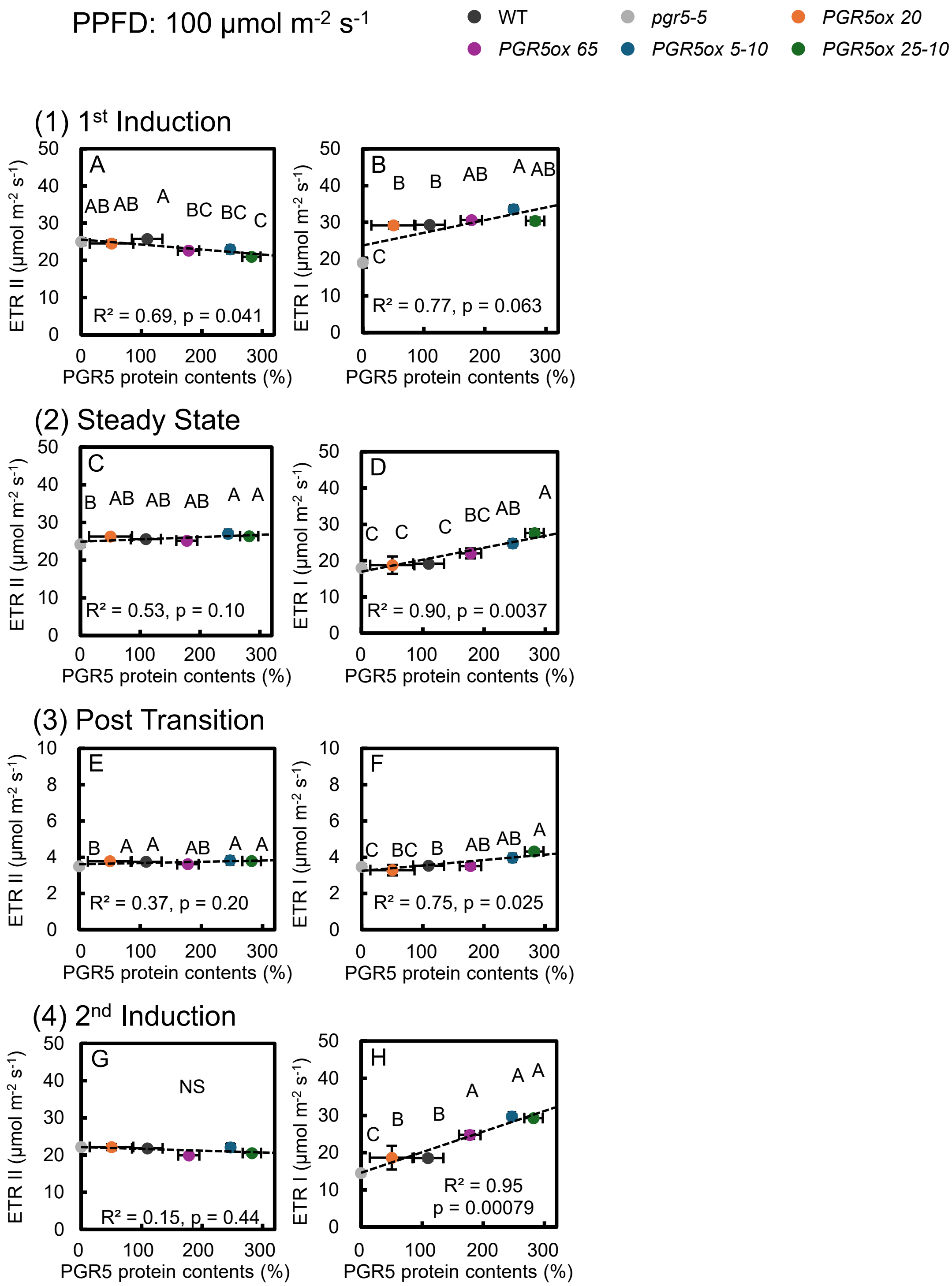

PPFD: 100 µmol m-2 s-1
WT
pgr5-5
PGR5ox 20
PGR5ox 65
PGR5ox 5-10
PGR5ox 25-10
(1) 1st Induction
(2) Steady State
(3) Post Transition
(4) 2nd Induction
ETR II (µmol m-2 s-1)
ETR I (µmol m-2 s-1)
PGR5 protein contents (%)
A
AB AB A BC BC C
R² = 0.69, p = 0.041
B
B B AB A AB
C
R² = 0.77, p = 0.063
C
B AB AB AB A A
R² = 0.53, p = 0.10
D
C C C BC AB A
R² = 0.90, p = 0.0037
E
B A A AB A A
R² = 0.37, p = 0.20
F
C BC B AB AB A
R² = 0.75, p = 0.025
G
NS
R² = 0.15, p = 0.44
H
C B B A A A
R² = 0.95
p = 0.00079
