# Supplementary material for: Moderate overexpression of PROTON GRADIENT REGULATION 5 improves photosynthetic performance and plant growth under fluctuating low light in Arabidopsis thaliana
Source: Plant J. 2026 Sep 28;127(6):e71120. doi: 10.1111/tpj.71120 (PMC13620446; doi:10.1111/tpj.71120)
Supplement: Supplementary file 10 [file TPJ-127-0-s010.pdf]

Figure S8

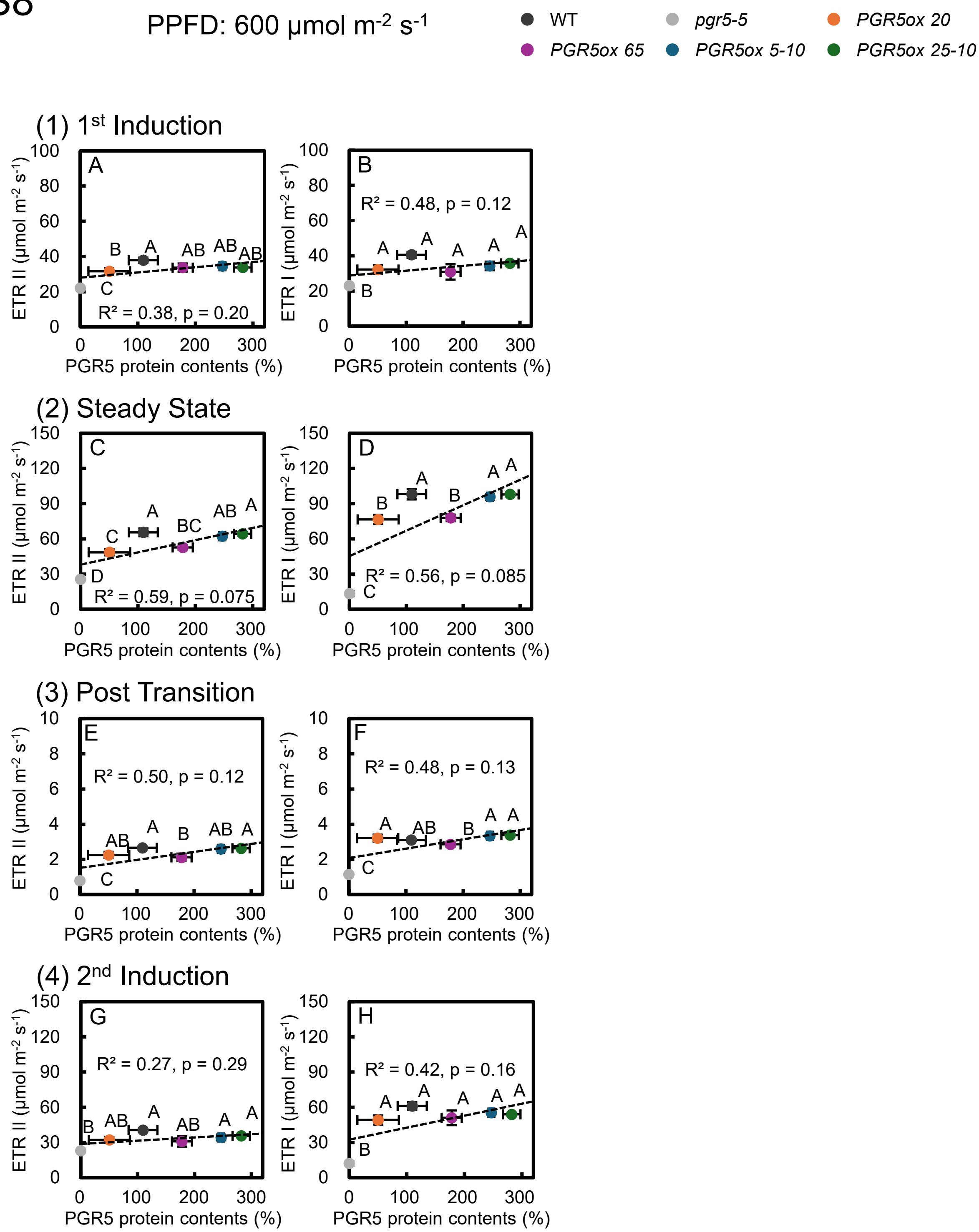
PPFD: 600 μmol m-2 s-1
WT
pgr5-5
PGR5ox 20
PGR5ox 65
PGR5ox 5-10
PGR5ox 25-10
(1) 1st Induction
A
R² = 0.38, p = 0.20
B
R² = 0.48, p = 0.12
(2) Steady State
C
R² = 0.59, p = 0.075
D
R² = 0.56, p = 0.085
(3) Post Transition
E
R² = 0.50, p = 0.12
F
R² = 0.48, p = 0.13
(4) 2nd Induction
G
R² = 0.27, p = 0.29
H
R² = 0.42, p = 0.16
ETR II (μmol m-2 s-1)
ETR I (μmol m-2 s-1)
PGR5 protein contents (%)
