# Supplementary material for: Moderate overexpression of PROTON GRADIENT REGULATION 5 improves photosynthetic performance and plant growth under fluctuating low light in Arabidopsis thaliana
Source: Plant J. 2026 Sep 28;127(6):e71120. doi: 10.1111/tpj.71120 (PMC13620446; doi:10.1111/tpj.71120)
Supplement: Supplementary file 11 [file TPJ-127-0-s011.pdf]

# Figure S10

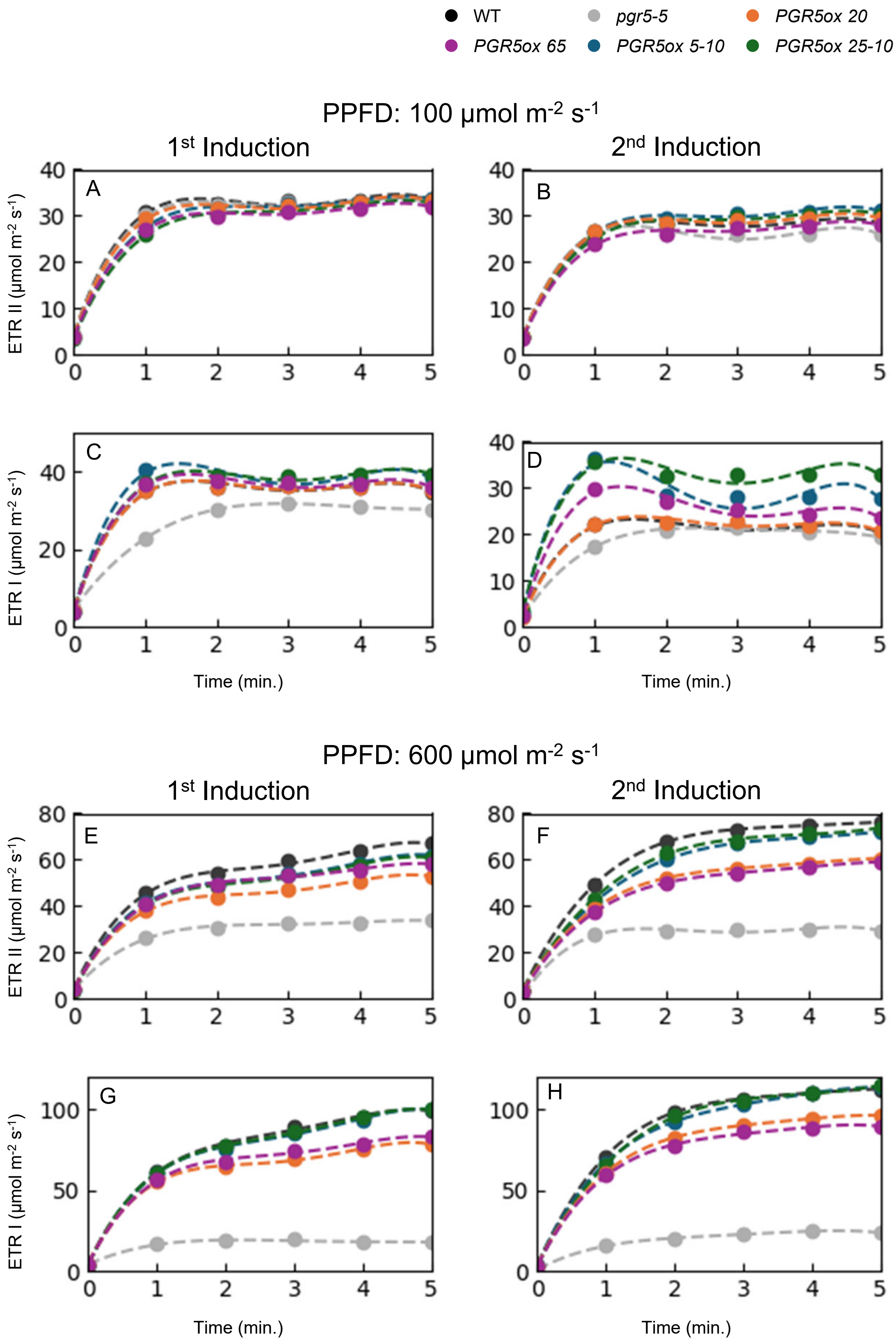

WT
pgr5-5
PGR5ox 20
PGR5ox 65
PGR5ox 5-10
PGR5ox 25-10
PPFD: 100 µmol m-2 s-1
1st Induction
2nd Induction
A
B
C
D
ETR II (µmol m-2 s-1)
ETR I (µmol m-2 s-1)
Time (min.)
PPFD: 600 µmol m-2 s-1
1st Induction
2nd Induction
E
F
G
H
ETR II (µmol m-2 s-1)
ETR I (µmol m-2 s-1)
Time (min.)
