# Supplementary material for: Moderate overexpression of PROTON GRADIENT REGULATION 5 improves photosynthetic performance and plant growth under fluctuating low light in Arabidopsis thaliana
Source: Plant J. 2026 Sep 28;127(6):e71120. doi: 10.1111/tpj.71120 (PMC13620446; doi:10.1111/tpj.71120)
Supplement: Supplementary file 13 [file TPJ-127-0-s013.pdf]

Figure S14

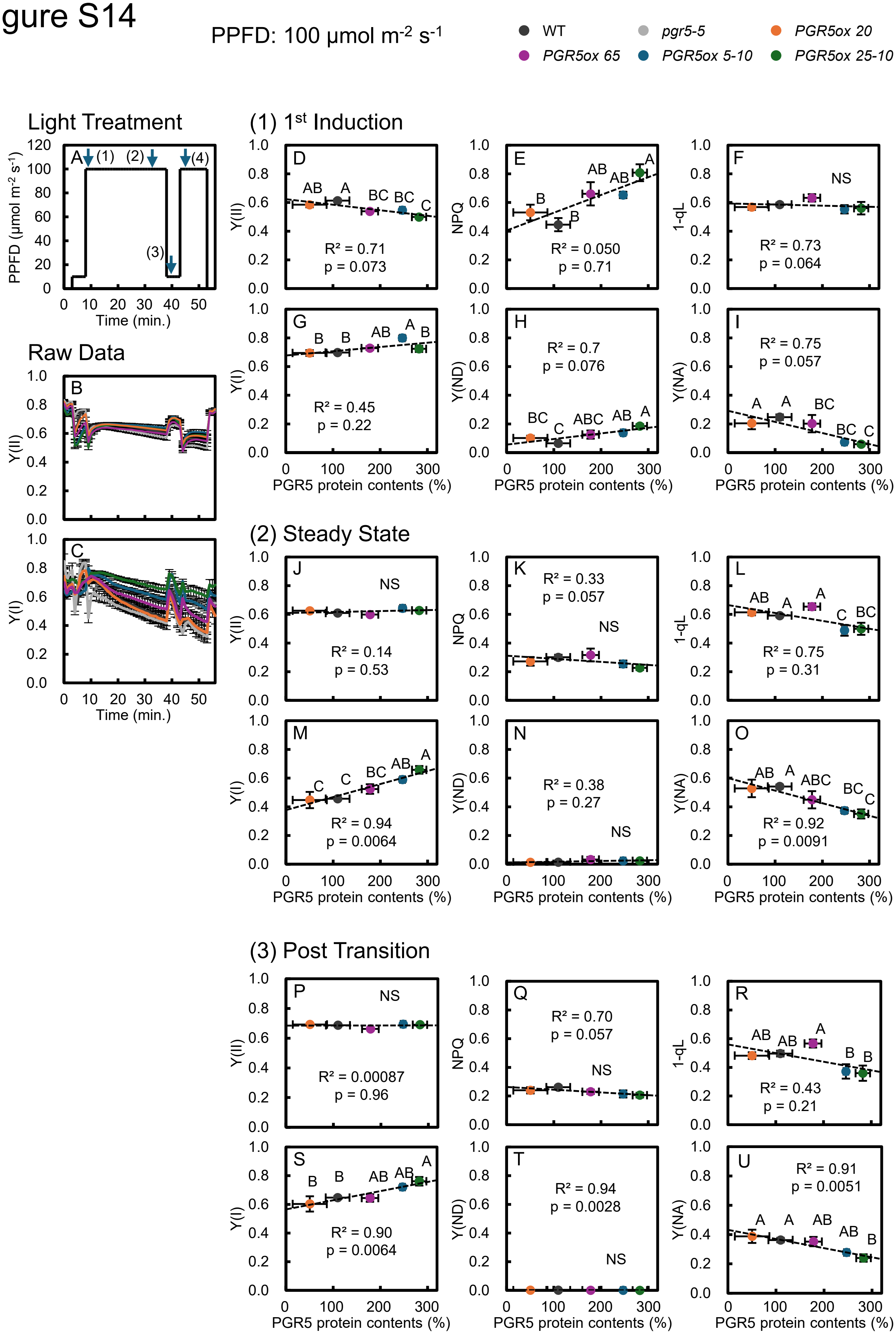

PPFD: 100 μmol m-2 s-1
WT
pgr5-5
PGR5ox 20
PGR5ox 65
PGR5ox 5-10
PGR5ox 25-10
Light Treatment
A
(1)
(2)
(3)
(4)
PPFD (μmol m-2 s-1)
Time (min.)
Raw Data
B
C
Y(II)
Y(I)
(1) 1st Induction
D
R² = 0.71
p = 0.073
E
R² = 0.050
p = 0.71
F
NS
R² = 0.73
p = 0.064
G
R² = 0.45
p = 0.22
H
R² = 0.7
p = 0.076
I
R² = 0.75
p = 0.057
(2) Steady State
J
NS
R² = 0.14
p = 0.53
K
R² = 0.33
p = 0.057
NS
L
R² = 0.75
p = 0.31
M
R² = 0.94
p = 0.0064
N
R² = 0.38
p = 0.27
NS
O
R² = 0.92
p = 0.0091
(3) Post Transition
P
NS
R² = 0.00087
p = 0.96
Q
R² = 0.70
p = 0.057
NS
R
R² = 0.43
p = 0.21
S
R² = 0.90
p = 0.0064
T
R² = 0.94
p = 0.0028
NS
U
R² = 0.91
p = 0.0051
NPQ
1-qL
Y(ND)
Y(NA)
PGR5 protein contents (%)
