# Supplementary material for: Moderate overexpression of PROTON GRADIENT REGULATION 5 improves photosynthetic performance and plant growth under fluctuating low light in Arabidopsis thaliana
Source: Plant J. 2026 Sep 28;127(6):e71120. doi: 10.1111/tpj.71120 (PMC13620446; doi:10.1111/tpj.71120)
Supplement: Supplementary file 14 [file TPJ-127-0-s014.pdf]

Figure S17

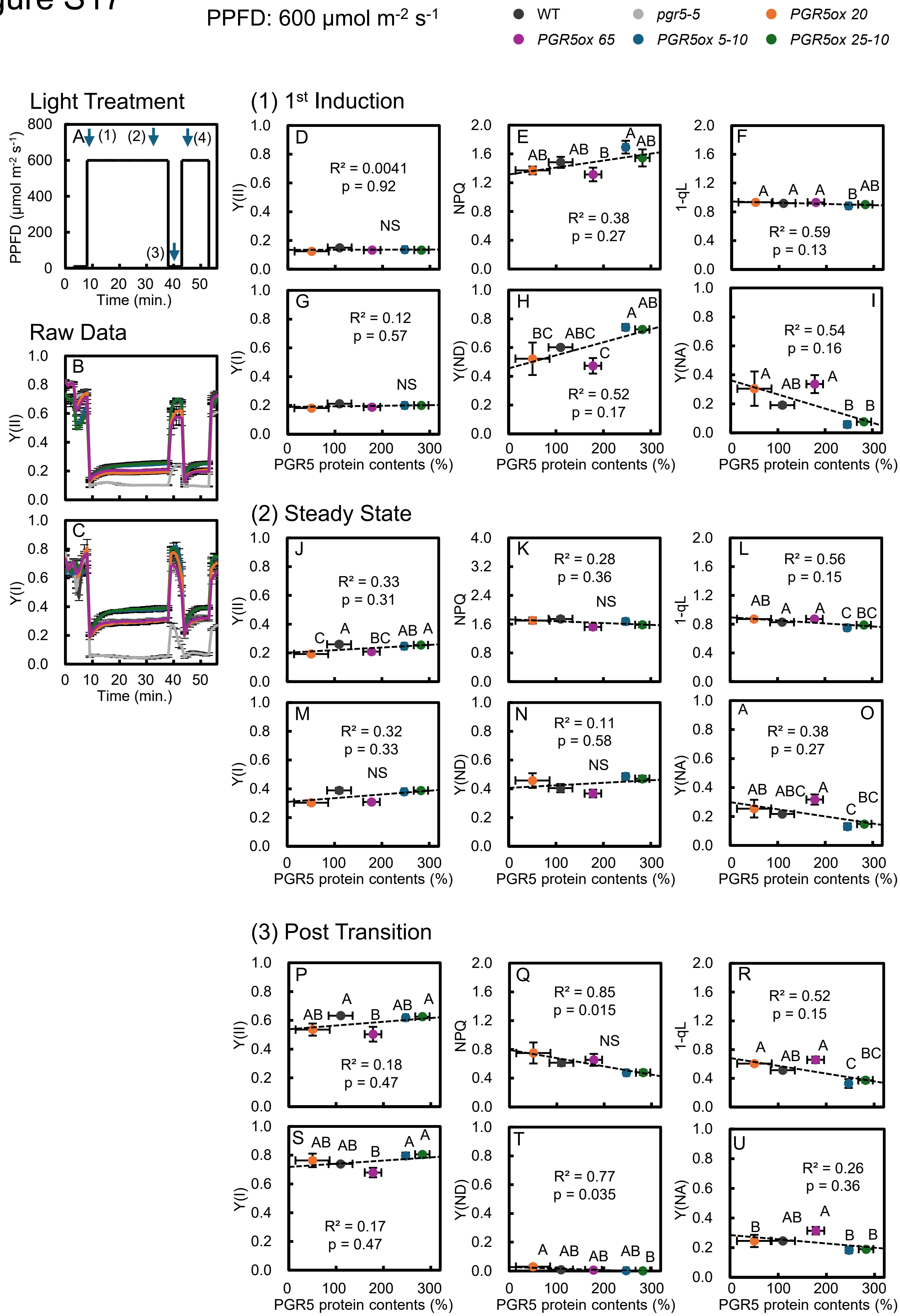

PPFD: 600 μmol m-2 s-1
WT
pgr5-5
PGR5ox 20
PGR5ox 65
PGR5ox 5-10
PGR5ox 25-10
Light Treatment
(1) 1st Induction
(2) Steady State
(3) Post Transition
Raw Data
PPFD (μmol m-2 s-1)
Time (min.)
PGR5 protein contents (%)
Y(II)
Y(I)
NPQ
1-qL
Y(ND)
Y(NA)
R² = 0.0041
p = 0.92
R² = 0.38
p = 0.27
R² = 0.59
p = 0.13
R² = 0.12
p = 0.57
R² = 0.52
p = 0.17
R² = 0.54
p = 0.16
R² = 0.33
p = 0.31
R² = 0.28
p = 0.36
R² = 0.56
p = 0.15
R² = 0.32
p = 0.33
R² = 0.11
p = 0.58
R² = 0.38
p = 0.27
R² = 0.18
p = 0.47
R² = 0.85
p = 0.015
R² = 0.52
p = 0.15
R² = 0.17
p = 0.47
R² = 0.77
p = 0.035
R² = 0.26
p = 0.36
NS
