# Supplementary material for: Moderate overexpression of PROTON GRADIENT REGULATION 5 improves photosynthetic performance and plant growth under fluctuating low light in Arabidopsis thaliana
Source: Plant J. 2026 Sep 28;127(6):e71120. doi: 10.1111/tpj.71120 (PMC13620446; doi:10.1111/tpj.71120)
Supplement: Supplementary file 16 [file TPJ-127-0-s016.pdf]

Figure S21

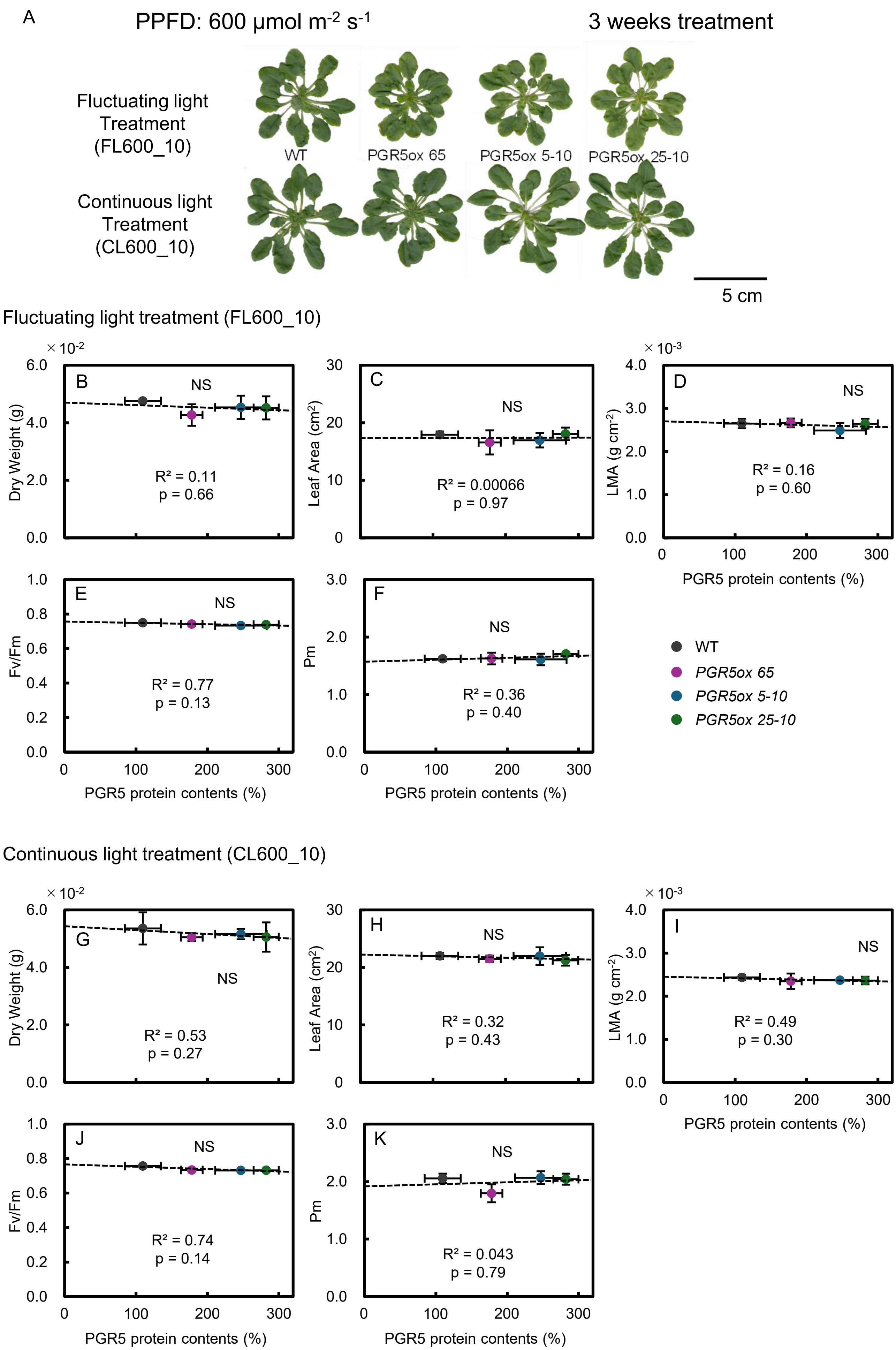

A
PPFD: 600 µmol m-2 s-1
3 weeks treatment
Fluctuating light Treatment (FL600_10)
Continuous light Treatment (CL600_10)
WT
PGR5ox 65
PGR5ox 5-10
PGR5ox 25-10
5 cm
Fluctuating light treatment (FL600_10)
B
C
D
E
F
NS
R² = 0.11
p = 0.66
R² = 0.00066
p = 0.97
R² = 0.16
p = 0.60
R² = 0.77
p = 0.13
R² = 0.36
p = 0.40
Dry Weight (g)
× 10-2
Leaf Area (cm2)
LMA (g cm-2)
× 10-3
Fv/Fm
Pm
PGR5 protein contents (%)
WT
PGR5ox 65
PGR5ox 5-10
PGR5ox 25-10
Continuous light treatment (CL600_10)
G
H
I
J
K
R² = 0.53
p = 0.27
R² = 0.32
p = 0.43
R² = 0.49
p = 0.30
R² = 0.74
p = 0.14
R² = 0.043
p = 0.79
