# Supplementary material for: Moderate overexpression of PROTON GRADIENT REGULATION 5 improves photosynthetic performance and plant growth under fluctuating low light in Arabidopsis thaliana
Source: Plant J. 2026 Sep 28;127(6):e71120. doi: 10.1111/tpj.71120 (PMC13620446; doi:10.1111/tpj.71120)
Supplement: Supplementary file 17 [file TPJ-127-0-s017.pdf]

Figure S18

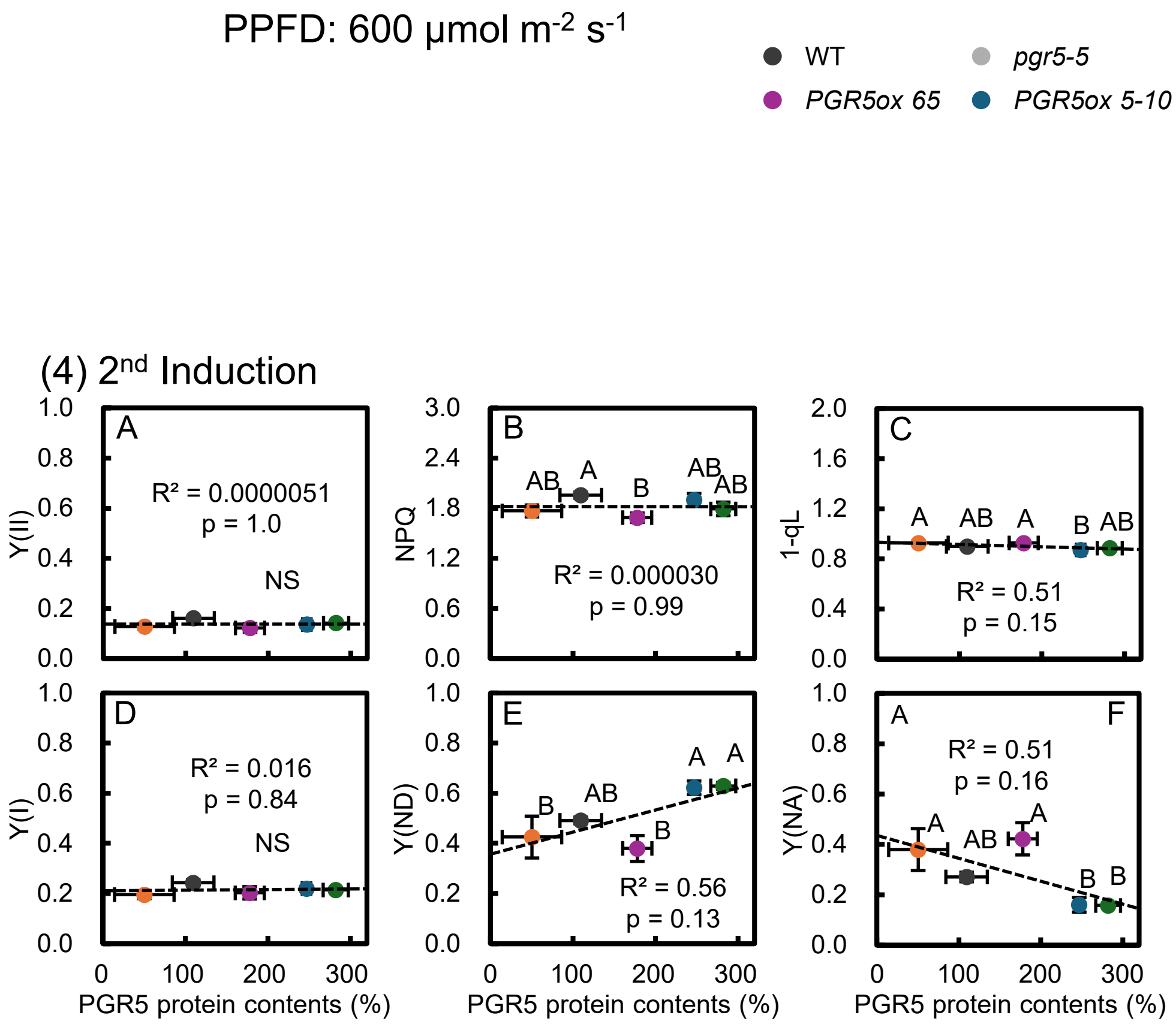
PPFD: 600 µmol m-2 s-1
WT
pgr5-5
PGR5ox 20
PGR5ox 65
PGR5ox 5-10
PGR5ox 25-10
(4) 2nd Induction
A
R² = 0.0000051
p = 1.0
NS
Y(II)
B
NPQ
R² = 0.000030
p = 0.99
C
1-qL
R² = 0.51
p = 0.15
D
Y(I)
R² = 0.016
p = 0.84
NS
E
Y(ND)
R² = 0.56
p = 0.13
F
Y(NA)
R² = 0.51
p = 0.16
PGR5 protein contents (%)
PGR5 protein contents (%)
PGR5 protein contents (%)
