# Supplementary material for: Moderate overexpression of PROTON GRADIENT REGULATION 5 improves photosynthetic performance and plant growth under fluctuating low light in Arabidopsis thaliana
Source: Plant J. 2026 Sep 28;127(6):e71120. doi: 10.1111/tpj.71120 (PMC13620446; doi:10.1111/tpj.71120)
Supplement: Supplementary file 18 [file TPJ-127-0-s018.pdf]

Figure S1

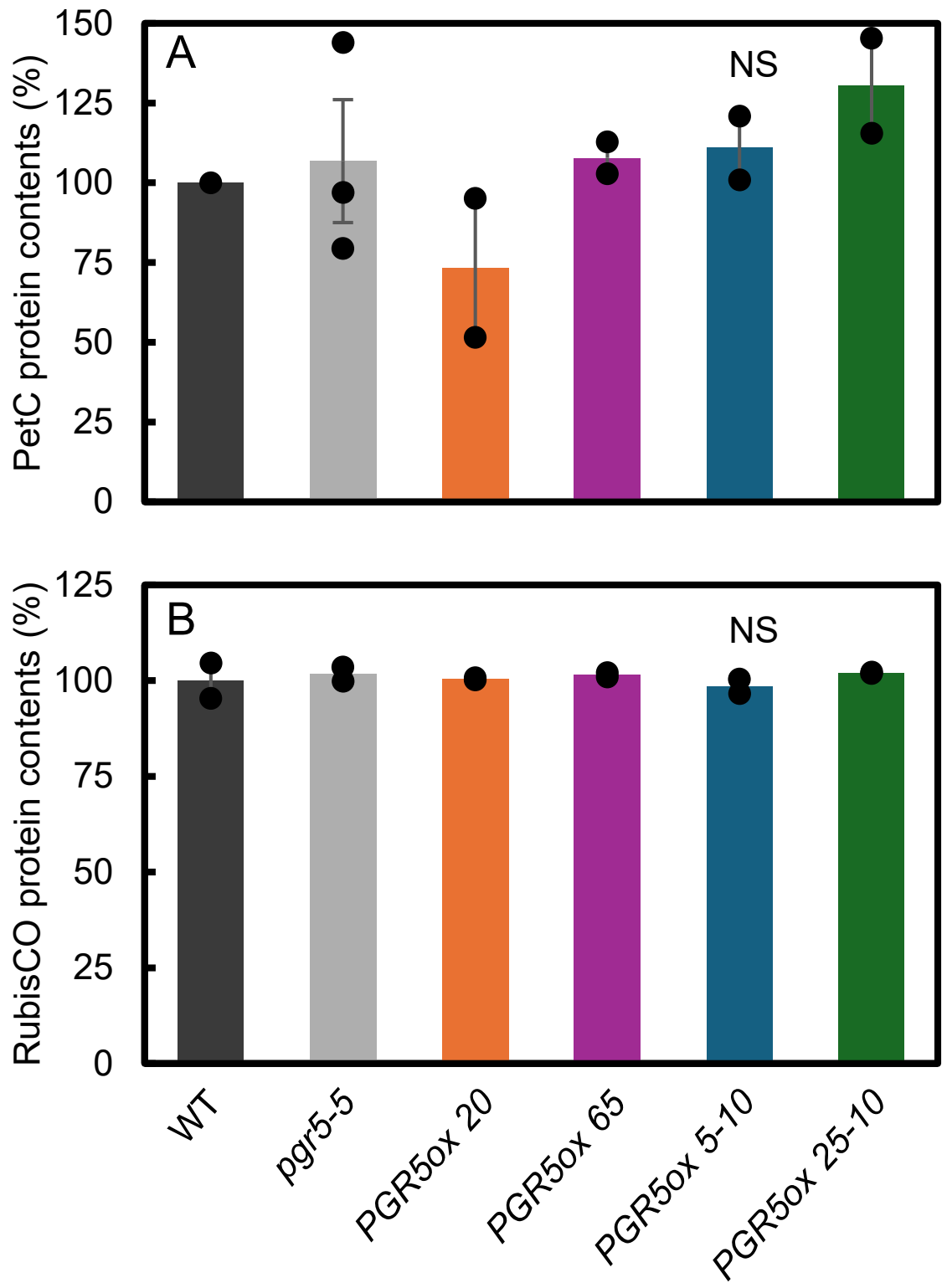

A
PetC protein contents (%)
150
125
100
75
50
25
0
NS
B
RubisCO protein contents (%)
125
100
75
50
25
0
NS
WT
pgr5-5
PGR5ox 20
PGR5ox 65
PGR5ox 5-10
PGR5ox 25-10
