# Supplementary material for: Moderate overexpression of PROTON GRADIENT REGULATION 5 improves photosynthetic performance and plant growth under fluctuating low light in Arabidopsis thaliana
Source: Plant J. 2026 Sep 28;127(6):e71120. doi: 10.1111/tpj.71120 (PMC13620446; doi:10.1111/tpj.71120)
Supplement: Supplementary file 19 [file TPJ-127-0-s019.pdf]

Figure S13

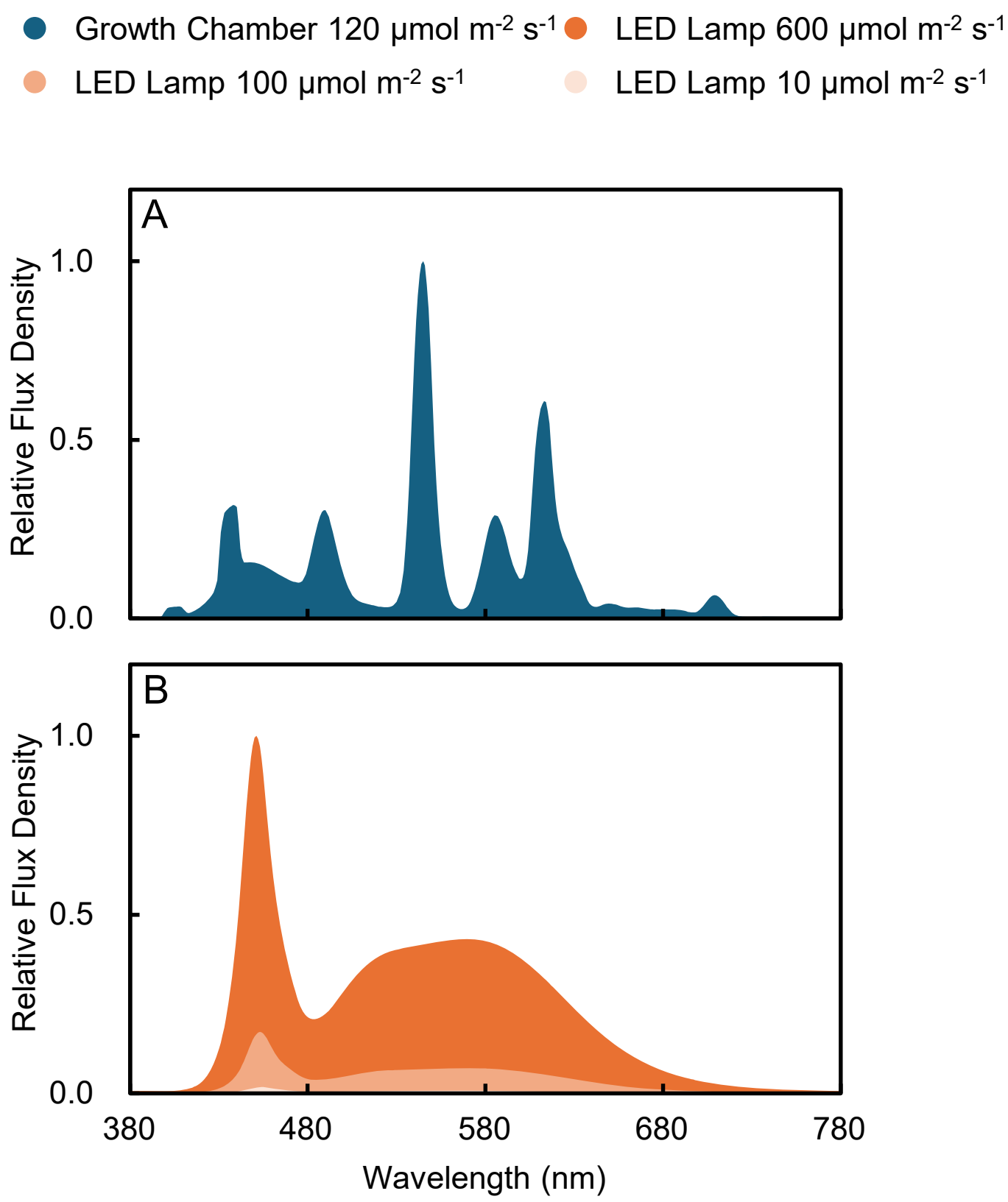
Growth Chamber 120 µmol m-2 s-1
LED Lamp 600 µmol m-2 s-1
LED Lamp 100 µmol m-2 s-1
LED Lamp 10 µmol m-2 s-1
A
B
Relative Flux Density
1.0
0.5
0.0
380
480
580
680
780
Wavelength (nm)
