# Supplementary material for: Moderate overexpression of PROTON GRADIENT REGULATION 5 improves photosynthetic performance and plant growth under fluctuating low light in Arabidopsis thaliana
Source: Plant J. 2026 Sep 28;127(6):e71120. doi: 10.1111/tpj.71120 (PMC13620446; doi:10.1111/tpj.71120)
Supplement: Supplementary file 20 [file TPJ-127-0-s020.pdf]

Figure S9

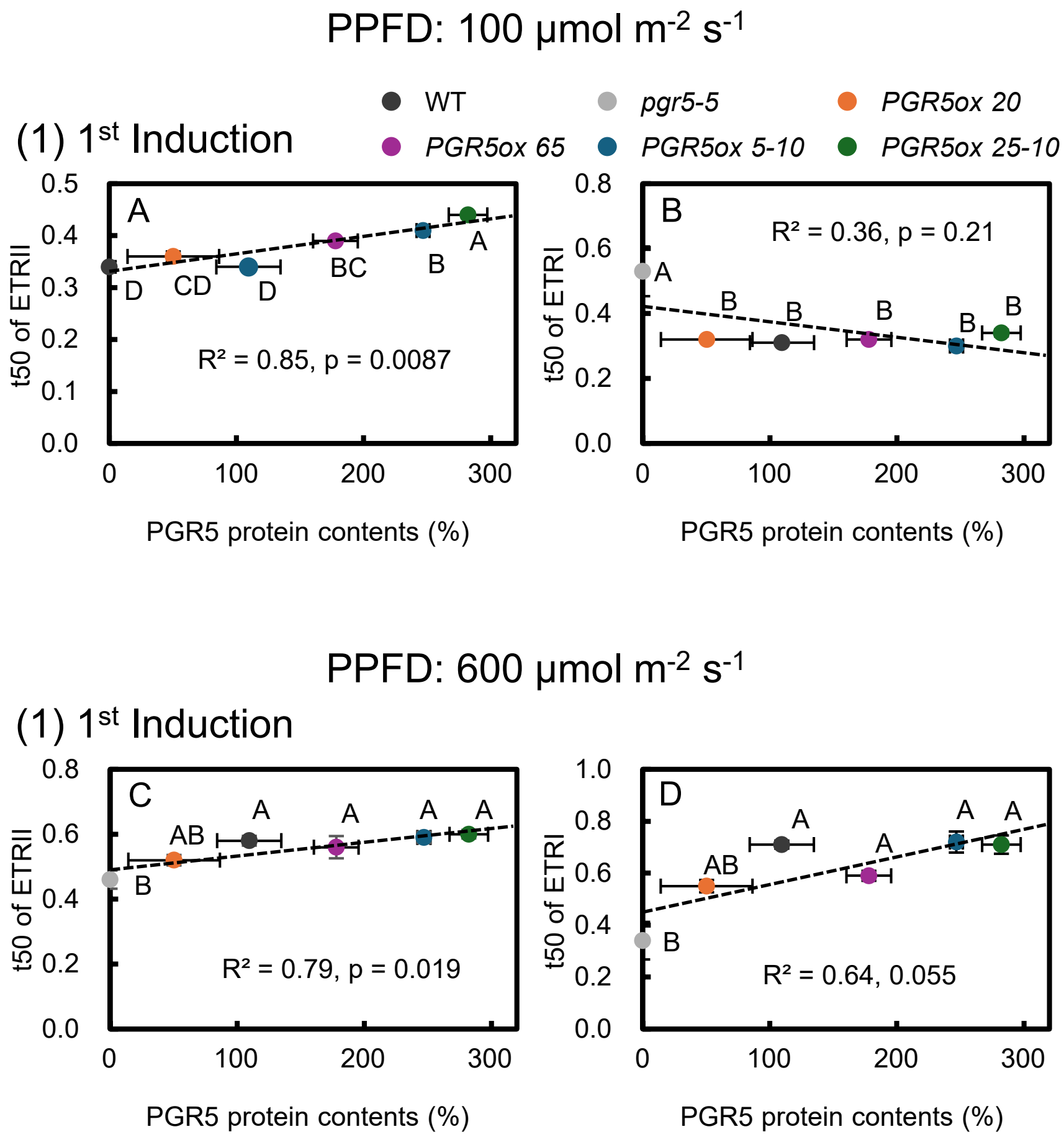

PPFD: 100 µmol m-2 s-1
(1) 1st Induction
WT
pgr5-5
PGR5ox 20
PGR5ox 65
PGR5ox 5-10
PGR5ox 25-10
A
R² = 0.85, p = 0.0087
D
CD
D
BC
B
A
t50 of ETRII
PGR5 protein contents (%)
B
R² = 0.36, p = 0.21
A
B
B
B
B
B
t50 of ETRI
PGR5 protein contents (%)
PPFD: 600 µmol m-2 s-1
(1) 1st Induction
C
R² = 0.79, p = 0.019
AB
A
A
A
A
B
t50 of ETRII
PGR5 protein contents (%)
D
R² = 0.64, 0.055
A
AB
A
A
A
B
t50 of ETRI
PGR5 protein contents (%)
