# Supplementary material for: Moderate overexpression of PROTON GRADIENT REGULATION 5 improves photosynthetic performance and plant growth under fluctuating low light in Arabidopsis thaliana
Source: Plant J. 2026 Sep 28;127(6):e71120. doi: 10.1111/tpj.71120 (PMC13620446; doi:10.1111/tpj.71120)
Supplement: Supplementary file 21 [file TPJ-127-0-s021.pdf]

Figure S4

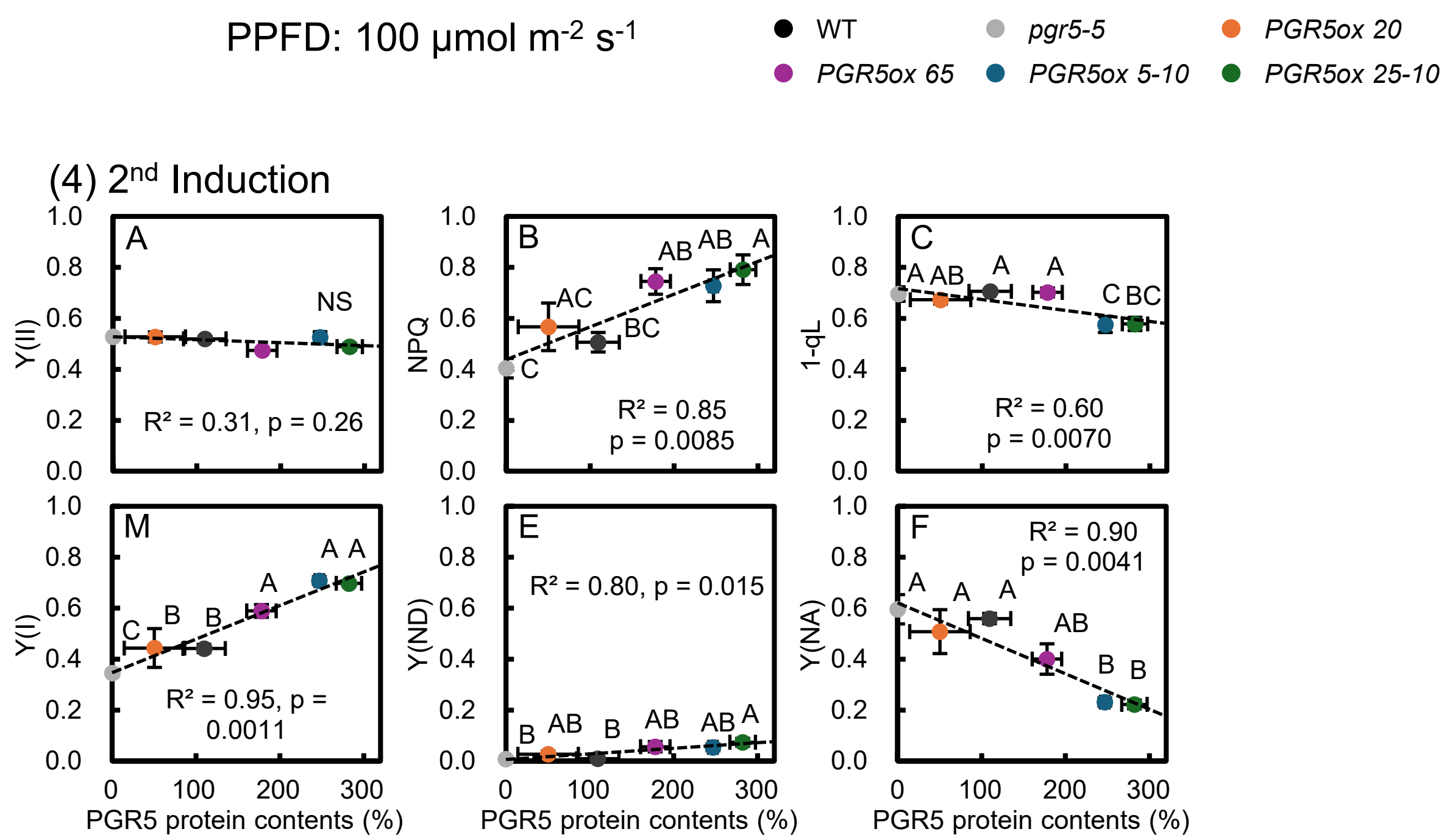
PPFD: 100 µmol m-2 s-1
WT
pgr5-5
PGR5ox 20
PGR5ox 65
PGR5ox 5-10
PGR5ox 25-10
(4) 2nd Induction
A
Y(II)
NS
R² = 0.31, p = 0.26
B
NPQ
A AB AB AC BC C
R² = 0.85
p = 0.0085
C
1-qL
A AB A A C BC
R² = 0.60
p = 0.0070
M
Y(I)
A A A C B B
R² = 0.95, p =
0.0011
E
Y(ND)
R² = 0.80, p = 0.015
B AB B AB AB A
F
Y(NA)
R² = 0.90
p = 0.0041
A A A AB B B
PGR5 protein contents (%)
